# An Unusual Skeletal Rearrangement in the Biosynthesis of the Sesquiterpene Trichobrasilenol from *Trichoderma*

**DOI:** 10.1002/anie.201907964

**Published:** 2019-09-09

**Authors:** Keiichi Murai, Lukas Lauterbach, Kazuya Teramoto, Zhiyang Quan, Lena Barra, Tsuyoshi Yamamoto, Kenichi Nonaka, Kazuro Shiomi, Makoto Nishiyama, Tomohisa Kuzuyama, Jeroen S. Dickschat

**Affiliations:** ^1^ Graduate School of Agricultural and Life Sciences The University of Tokyo 1-1-1 Yayoi, Bunkyu-ku Tokyo 113-8657 Japan; ^2^ Kekulé-Institute for Organic Chemistry and Biochemistry University of Bonn Gerhard-Domagk-Straße 1 53121 Bonn Germany; ^3^ Biotechnology Research Center The University of Tokyo 1-1-1 Yayoi, Bunkyu-ku Tokyo 113-8657 Japan; ^4^ Kitasato Institute for Life Sciences Kitasato University 5-9-1 Shirokane, Minato-ku Tokyo 108-8641 Japan; ^5^ Collaborative Research Institute for Innovative Microbiology The University of Tokyo 1-1-1 Yayoi, Bunkyu-ku Tokyo 113-8657 Japan

**Keywords:** biosynthesis, enzyme mechanisms, isotopes, NMR spectroscopy, terpenes

## Abstract

The skeletons of some classes of terpenoids are unusual in that they contain a larger number of Me groups (or their biosynthetic equivalents such as olefinic methylene groups, hydroxymethyl groups, aldehydes, or carboxylic acids and their derivatives) than provided by their oligoprenyl diphosphate precursor. This is sometimes the result of an oxidative ring‐opening reaction at a terpene‐cyclase‐derived molecule containing the regular number of Me group equivalents, as observed for picrotoxan sesquiterpenes. In this study a sesquiterpene cyclase from *Trichoderma* spp. is described that can convert farnesyl diphosphate (FPP) directly via a remarkable skeletal rearrangement into trichobrasilenol, a new brasilane sesquiterpene with one additional Me group equivalent compared to FPP. A mechanistic hypothesis for the formation of the brasilane skeleton is supported by extensive isotopic labelling studies.

## Graphical abstract

**Out of the ordinary**: Trichobrasilenol was identified as an unusual sesquiterpene alcohol made by a sesquiterpene cyclase from *Trichoderma* spp. The mechanism of product formation was studied by using isotopically labelled substrates, with a special emphasis on a complex rearrangement step that leads to the extrusion of a hydroxymethyl group from an internal methylene group of FPP.

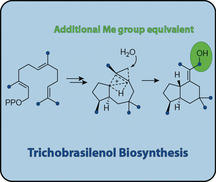

With an estimated number of more than 80,000 different compounds, terpenoids are the largest class of natural products.^[1]^ Their basic carbon skeletons derive from just a few acyclic precursors made through regular coupling of the monomers dimethylallyl diphosphate (DMAPP) and isopentenyl diphosphate (IPP), including geranyl (GPP), farnesyl (FPP), geranylgeranyl (GGPP), and (GFPP) geranylfarnesyl diphosphate. These precursor molecules are converted by the action of terpene cyclases (TCs) that ionise their substrate through diphosphate abstraction or protonation.[^1^, ^2^, ^3^, ^4^, ^5^] The inherent reactivity of the initially formed cation usually leads to a polycyclic skeleton with several stereogenic centres.^[6]^ In these reactions, the main tasks of the enzyme are to control the substrate conformation, to trigger the start of the reaction by the ionisation step, to provide a hydrophobic cavity essentially free of water to prevent premature cation quenchings, and to terminate the reaction through a strictly controlled deprotonation or addition of water to the latest cationic intermediate. For many of the known polycyclic skeletons, a possible mechanism of formation from the corresponding precursor seems obvious, but sometimes only isotopic labelling experiments can unravel surprising rearrangement steps or are required to distinguish between different alternatives.[^7^, ^8^, ^9^] The diversity of skeletons is further enlarged by irregular couplings of DMAPP and IPP that can help to explain unusual terpene skeletons,^[10]^ but in other cases, skeletal rearrangements of an acyclic (regular) precursor must be assumed. Particularly interesting are systems in which the number of Me groups or their equivalents such as olefinic methylene groups exceeds the number of Me groups in the precursor. For example, the picrotoxan sesquiterpene alkaloid dendrobine (**1**) from *Dendrobium nobile*,[^11^, ^12^] a compound with antipyretic, hypertensive, and convulsant activity, contains five such Me group equivalents (Figure 1). Its formation from FPP, which contains four Me groups, has been demonstrated by the incorporation of labelling from tritiated farnesol fed to *D. nobile*.^[13]^ The conversion of regiospecifically labelled copaborneol (**2**) into tutin (**3**) by *Coriaria japonica* demonstrated that the additional Me group equivalent in picrotoxans is the result of a ring opening reaction of **2** in a post‐TC step,^[14]^ while **2** itself can be rationalised as a regular sesquiterpene cyclase (STC) product (Scheme S1 in the Supporting Information).

**Figure 1:**
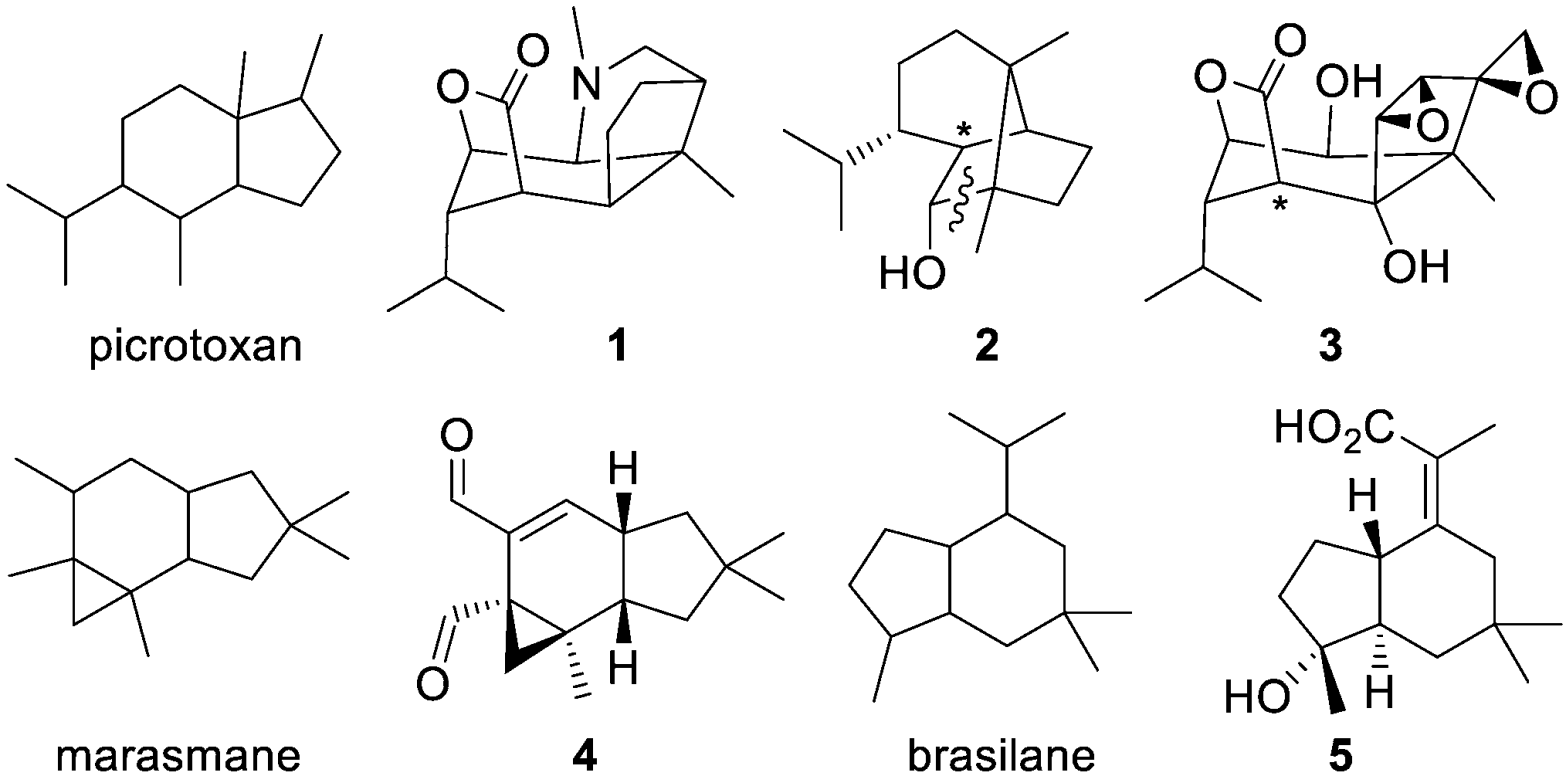
Examples of sesquiterpenoids with five Me group equivalents.

The marasmane derivative isovelleral (**4**) also contains five Me group equivalents and is a strongly antifungal and antibacterial sesquiterpenoid with a pungent taste from the fungus *Lactarius vellereus*,^[15]^ but the biogenesis of **4** or related compounds of the marasmane type has not been investigated so far. Brasilane sesquiterpenes such as the antibacterial compound xylarenic acid (**5**) also exhibit an extra Me group equivalent compared to FPP.^[16]^ We have now addressed the intricate and, despite all the accumulated work on terpene biosynthesis, still unsolved mechanistic problem of the formation of such additional Me groups by the identification of a brasilane STC from *Trichoderma* spp. that directly extrudes a fifth Me group equivalent from the FPP chain during the terpene cyclisation cascade.

The genome of *Trichoderma atroviride* FKI‐3849^[17]^ was sequenced and found to encode at least seven type I TCs (named TaTC1–7). The gene of one of these enzymes (TaTC6, accession number LC484924) that is not clustered with other biosynthetic genes and whose gene product is phylogenetically distant from other characterised fungal TCs (Figure S1 in the Supporting Information) was cloned and heterologously expressed in *Aspergillus oryzae*, resulting in the formation of a new product (Figure S2). The purified protein obtained by heterologous expression in *Escherichia coli* BL21(DE3) (Figure S3) exhibited all highly conserved motifs that are required for substrate binding and functionality (Figures S4 and S5) and converted FPP into a mixture of sesquiterpene hydrocarbons and alcohols (Figure S6), while GPP, GGPP, and GFPP were not accepted. The main product was purified by silica column chromatography and identified by extensive NMR spectroscopy (Table S3 and Figures S7–S13) as a new sesquiterpene alcohol for which we suggest the name trichobrasilenol (**6**, Scheme 1 A). The phylogenetic tree (Figure S1) shows that closely related enzymes are present in several other *Trichoderma* strains (see also Figure S14), and heterologous expression of the corresponding gene from *Trichoderma reesei* QM6a in *Aspergillus oryzae* yielded the same compound **6** (Figure S15), suggesting the same function for all these related enzymes.

**Scheme 1:**
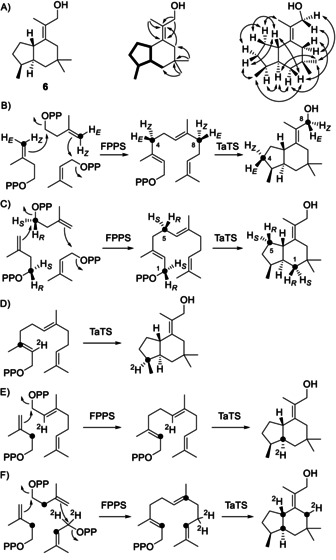
Trichobrasilenol (**6**). A) Structure elucidation (bold: COSY, single headed arrows: key HMBC, double headed arrows: key NOESY correlations). B,C) Isotopic labelling experiments to solve the absolute configuration of **6**. D–F) Isotopic labelling experiments to follow hydride shifts in the biosynthesis of **6**.

The absolute configuration of **6** was resolved using enantioselectively deuterated FPP isotopomers. Their conversion with TaTC6 resulted in stereoselectively deuterated carbons of **6** with known configuration. The absolute configuration was then correlated by solving the relative orientation of the other stereocentres to the incorporated deuterium. An additional ^13^C label at the deuterated carbon enabled sensitive HSQC analysis of the products obtained from small scale reactions without the need for compound purification. Specifically, (*E*)‐ and (*Z*)‐(4‐^13^C,4‐^2^H)IPP^[18]^ were used to elongate DMAPP using the FPP synthase (FPPS) from *Streptomyces coelicolor* A3(2),^[19]^ followed by cyclisation with TaTC6, thus resulting in the incorporation of labelling at C4 and C8 (Scheme 1 B). The HSQC spectra of the labelled products (Figure 2) together with the assignments of the diastereotopic hydrogens at C4 based on the NOESY spectrum (Table S3 and Figure S13) pointed to the absolute configuration of **6** as shown in Scheme 1. Similarly, DMAPP was elongated with (*R*)‐ and (*S*)‐(1‐^13^C,1‐^2^H)IPP^[20]^ catalysed by FPPS for cyclisation by TaTC6 (Scheme 1 C). The HSQC spectra of the products labelled at C1 and C5 indicated the same absolute configuration for **6** (Figure S16). The interpretation of these experiments is based on the known stereochemical course for the addition of IPP to DMAPP and GPP in the elongation to FPP,^[21]^ and on the assumption of an inversion of configuration at C1 of FPP in the initial cyclisation, as observed for other TCs.^[22]^

**Figure 2:**
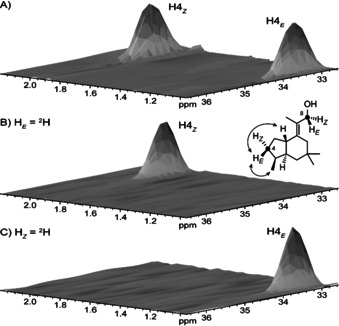
The absolute configuration of **6**. Partial HSQC spectra of A) unlabelled **6**, B) labelled **6** obtained from DMAPP and (*E*)‐(4‐^13^C,4‐^2^H)IPP, and C) labelled **6** obtained from DMAPP and (*Z*)‐(4‐^13^C,4‐^2^H)IPP (Scheme 1 B). Important NOE correlations for interpretation of the results are specified, additional NOE correlations are shown in Scheme 1.

Additionally, several side products were identified by GC/MS (Scheme 2 A, Table S5), including α‐humulene (**7**), caryophyllene (**8**), 2‐*epi*‐caryophyllene (**9**), african‐3‐ene (**10**) and african‐1‐ene (**11**), while isoafricanol (**12**) and pristinol (**13**) were identified by comparison to authentic standards obtained with recently characterised STCs from *Streptomyces malaysiensis* and *Streptomyces pristinaespiralis*.[^23^, ^24^] All these compounds are formed from FPP by an initial 1,11‐cyclisation, thus suggesting that this may also be the case for **6**. A proposed cyclisation mechanism for TaTC6 towards **6** (Scheme 2 B) starts from FPP with abstraction of diphosphate and 1,11‐cyclisation. To avoid secondary cations as intermediates, a concerted reaction with simultaneous 1,2‐hydride migration and double ring closure to **A** can be assumed, which is followed by another 1,2‐hydride shift to **B**. A third 1,2‐hydride transfer leads to **C** that can be attacked by water with a remarkable rearrangement through the transition state **D^≠^**, involving another simultaneous hydride shift, cyclopropane ring opening, ring contraction, and double bond installation, ultimately leading to **6**. The side product **7** can be explained by 1,11‐cyclisation of FPP with direct deprotonation from C9, while **8** and **9** require additional 2,10‐cyclisation. Compounds **10**–**13** may arise through very similar reactions as **6**, but their stereochemistry at C2 or C3 is different, requiring a different stereochemical course of the cyclisation cascade.

**Scheme 2:**
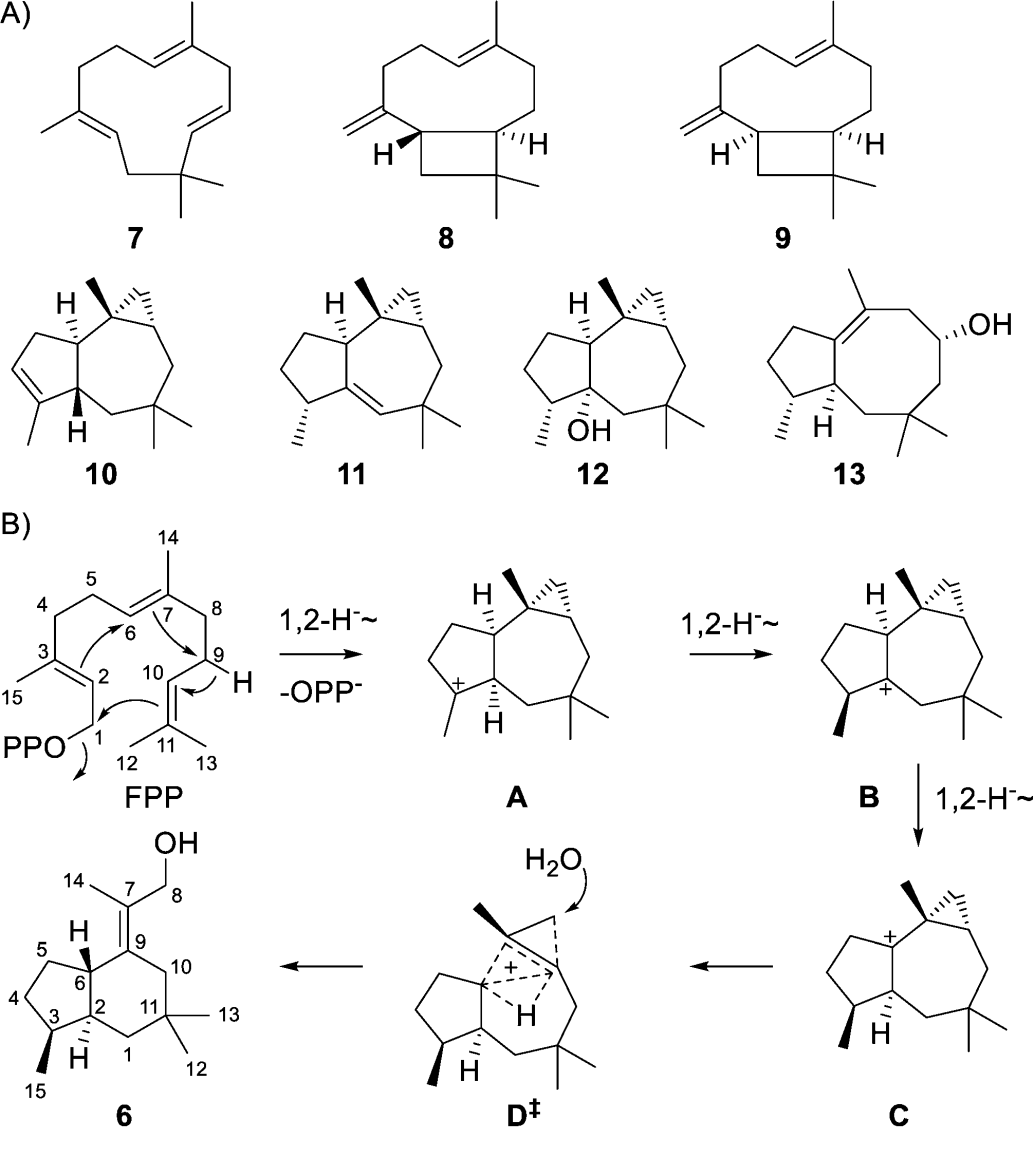
A) Identified side products of TaTC6. B) Cyclisation mechanism from FPP to **6**. Compound numbering for **6** shows the origin of each carbon from FPP.

This mechanistic hypothesis to explain the direct formation of a fifth Me group equivalent in a TC‐catalysed reaction was investigated in a series of isotopic‐labelling experiments. The incubation of all fifteen isotopomers of (^13^C)FPP^[25]^ with TaTC6 resulted in the incorporation of labelling into the expected positions in all cases (Figure S17), which especially supported the proposed rearrangement of the C6‐7‐8‐9‐10 portion of FPP in the step from **C** via **D^≠^** to **6**.

The hydride migration from **A** to **B** was followed by using (3‐^13^C,2‐^2^H)FPP,^[24]^ resulting in a slightly upfield‐shifted strong triplet for C3 of **6** in the ^13^C NMR spectrum (Scheme 1 D, Figure 3 A). The third hydride migration from **B** to **C** was investigated through the same strategy using (2‐^2^H)GPP^[26]^ and (2‐^13^C)IPP,^[27]^ which were coupled by FPPS to (2‐^13^C,6‐^2^H)FPP. Enzymatic conversion with TaTC6 yielded a product with a direct ^13^C−^2^H bond as indicated by a strongly enhanced triplet for C2 in the ^13^C‐NMR spectrum (Scheme 1 E, Figure 3 B). The remaining two hydride shifts from FPP to **A** and from **C** via **D^≠^** to **6** were addressed simultaneously in an experiment with (2‐^13^C,1,1‐^2^H_2_)DMAPP and (2‐^13^C)IPP, which were coupled by FPPS to yield (2,6,10‐^13^C_3_,9,9‐^2^H_2_)FPP. Its conversion by TaTC6 into labelled **6** yielded three enhanced signals for the labelled carbons (Scheme 1 F, Figure 3 C). The signal for C2 showed a very small upfield shift that is explainable by a deuterium at a neighbouring carbon and a doublet coupling with C6. The signal for C6 exhibited a typical upfield shift for a directly bound deuterium, which is also evident from the triplet coupling in addition to the doublet coupling with C2. Finally, C10 was also connected to deuterium as evident from the upfield shift and the triplet coupling. Taken together, these data supported both hydride shifts, from FPP to **A** and from **C** to **6**.

**Figure 3:**
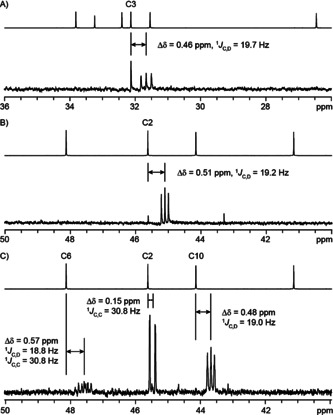
NMR spectra of the products obtained in labelling experiments to follow hydride shifts in the biosynthesis of **6**. Partial ^13^C‐NMR spectra of unlabelled **6** (top) and of labelled **6** (bottom) obtained from A) (3‐^13^C,2‐^2^H)FPP, B) (2‐^2^H)GPP and (2‐^13^C)IPP, and C) (2‐^13^C,1,1‐^2^H_2_)DMAPP and (2‐^13^C)IPP.

In summary, we have identified two representatives from a clade of STCs from *Trichoderma* that produce the new sesquiterpene alcohol trichobrasilenol with an additional Me group equivalent in comparison to FPP. The mechanism of its formation was addressed using isotopically labelled precursors, thus demonstrating the extrusion of this additional Me group equivalent in the form of a hydroxymethyl group from an internal methylene group of FPP in a notable rearrangement step along the cationic cyclisation cascade to **6**. This main finding of our study represents an unprecedented reaction in terpene biosynthesis, most likely in a concerted process, since there is no good explanation via a stepwise procedure (cf. Scheme S2 with a detailed discussion in the Supporting Information). A similar mechanism may also be relevant for the biosynthesis of sodorifen,^[28]^ a polymethylated homosesquiterpene in which one additional Me group equivalent is introduced by an SAM‐dependent methylation, but in this case, three more Me groups seem to originate from internal methylene groups of FPP.^[29]^ Despite preliminary results from labelling experiments that have resulted in a biosynthetic hypothesis,^[29]^ a detailed mechanistic investigation for the biosynthesis of this molecule is lacking. In the case of the homomonoterpene 2‐methylisoborneol, one additional Me group is introduced by SAM‐dependent methylation of GPP, but the subsequent terpene cyclisation of the resulting 2‐methyl‐GPP is regular.[^30^, ^31^, ^32^] While for homoterpenes, the presence of the additional Me group introduced by a methyltransferase is not surprising, the puzzling case of brasilane biosynthesis with an additional Me group equivalent was solved here. In conclusion, different mechanisms exist for the introduction of additional Me group equivalents into terpenes, and with this study we provide the first mechanistic investigation of a TC that catalyses carbon extrusion via a complex skeletal rearrangement.

## Conflict of interest

The authors declare no conflict of interest.

## Supporting information

As a service to our authors and readers, this journal provides supporting information supplied by the authors. Such materials are peer reviewed and may be re‐organized for online delivery, but are not copy‐edited or typeset. Technical support issues arising from supporting information (other than missing files) should be addressed to the authors.

Supplementary
