# Supplementary material for: An Unusual Skeletal Rearrangement in the Biosynthesis of the Sesquiterpene Trichobrasilenol from Trichoderma
Source: Angew Chem Int Ed Engl. 2019 Sep 9;58(42):15046–50. doi: 10.1002/anie.201907964 (PMC7687074; doi:10.1002/anie.201907964)
Supplement: Supplementary file 1 — Supplementary [file ANIE-58-15046-s001.pdf]

Angewandte Chemie
Eine Zeitschrift der Gesellschaft Deutscher Chemiker

Supporting Information

An Unusual Skeletal Rearrangement in the Biosynthesis of the Sesquiterpene Trichobrasilenol from *Trichoderma*

Keiichi Murai[+], Lukas Lauterbach[+], Kazuya Teramoto, Zhiyang Quan, Lena Barra, Tsuyoshi Yamamoto, Kenichi Nonaka, Kazuro Shiomi, Makoto Nishiyama, Tomohisa Kuzuyama, and Jeroen S. Dickschat**

anie_201907964_sm_miscellaneous_information.pdf

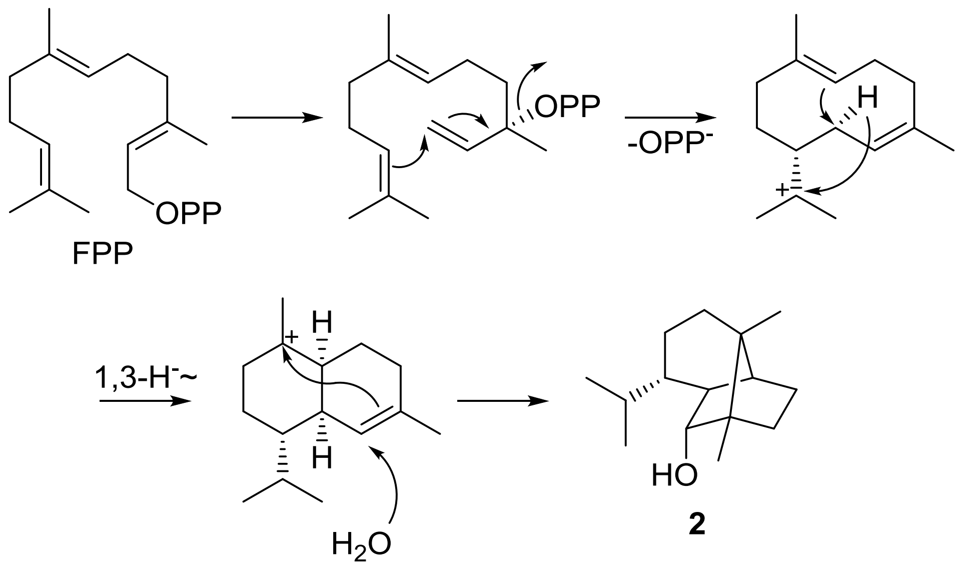

Scheme S1. Plausible biosynthetic pathway from FPP to copaborneol (**2**).

Table S1. Primers used in plasmid construction for *A. oryzae*.

Primer	Sequence (5' to 3')
*taTC6*_KpnI_Fw	AAAGGTACCATGGGTCAACCCACGACAACTAG[a]
*taTC6*_KpnI_Rv	TTTGGTACCAAACAGCCTACGAAGCGG[a]
RT_*taTC6*_Nhis_Fw	TGGTTCCCATGGCGGATCCGGTCAACCCACGACAACTAG
RT_*taTC6*_Nhis_Rv	CGAGTGCGGCCGCAAGCTTTCATGTACGCGATAAATCCAAC
TrTS_Fw	AGCAAGCTCCGAATTATGGACTCGCTATGGAGTATTGTCG
TrTS_Rv	TACCGAGCTCGAATTCTAACGGTAAGAGAGGTCCAACAGC
TEF_Tmn-Fw	CGGTACCCGGGGATCCGCTGGTTAATATGTGCGGGG
TEF_Tmn-Rv	TGATTACGCCAAGCTGTTGTCCGCAAGACGCTTTTC
TEF_Prm-Fw	AAACGACGGCCAGTGAATTTTGTTTTTTTGTTTTTTTGTTTTTTTTGTTCCTTG
TEF_Prm-Rv	GATCCCCGGGTACCGAGCTCTTTGAAGGTGGTGCGAACTTTG
FPPS_Fw	AAGAGCTCGGTACCCATGCCATCTACTACAAGCCGCGCTG
FPPS_Rv	AACCAGCGGATCCCCTTATTTACTGCGCTTGTAGATCTTCCCGAGGAATGCC
pUC19_Fw	GAACCTGGTATCCATGGCGTAATCATGGTCATAGCTG
pUC19_Rv	AGACAGTAAAAGATGGGTACCGAGCTCGAATTCACTG
AdeA_Fw	CATCTTTTACTGTCTACATCAACGGCG
AdeA_Rv	ATGGATACCAGGTTCAATTTGTATAGTAATTTATATTTTTAGG
TEF_2_AdeA-Fw	AACGACGGCCAGTGAATTCGAACATCTACATACTACGTAGATTGTCACCG
TEF_2_AdeA-Rv	CAGTAAAAGATGGGGTACCGGTCCGCAAGACGCTTTTCTGAC

[a] KpnI restriction sites are underlined.

Identification of terpene synthase genes in *Trichoderma atroviride* FKI-3849

Genomic DNA of *T. atroviride* FKI-3849 was prepared according to the CTAB method (see http://1000.fungalgenomes.org/home/wp-content/uploads/2013/02/genomicDNAProtocol-AK0511.pdf). Only the step using Qiagen Genome-tip 500/G was not done for further purification, because pulse field agarose gel electrophoresis showed that the quality of genomic DNA sample was sufficient for sequencing. Draft genome sequencing of *T. atroviride* FKI-3849 was carried out using the Genome Sequencer FLX System (Roche Diagnostics, Tokyo) at Takara Bio Inc, Tokyo. The genome size was estimated 35.4 Mb, and the draft assembly yielded an average contig length of 5.7 kb and a large number of 7200 contigs. The gene prediction program AUGUSTUS.2.5.5 (http://augustus.gobics.de/binaries/) was used to construct a putative ORF set from the draft genome sequence of *T. atroviride* FKI-3849. *Aspergillus oryzae* was adopted as a model for the ORF prediction. To retrieve uncharacterized terpene synthases, the hidden Markov model (HMM) with the software package hmmer-3.1b2 (http://hmmer.org/download.html) was used. For HMM generation characterized terpene synthases in a UniProtKB database (http://www.uniprot.org) were selected (Table S2). Their sequences were aligned using MAFFT (https://mafft.cbrc.jp/alignment/software/macosx.html). Based on the resulting alignment, an HMM was generated using a hmmbuild program included in the hmmer-3.1b2. The generated HMM was subjected to a "hmmpress" command, and then a "hmmscan" or "hmmsearch" command was performed to retrieve putative terpene synthases from the ORF set of *T. atroviride* FKI-3849. This search allowed us to find a putative terpene synthase (TaTC6) containing both a DDxxD motif and an NSE/DTE motif, with an E-value of 3.2×10^{-8}.

Construction of phylogenetic tree

For a phylogenetic analysis a BLAST search with the amino acid sequences of several characterised fungal terpene synthases as probes (accession numbers include Q6WP50, AAN05035, XP_023437750, Q9UR08, S0EA85) was performed to find fungal terpene cyclase homologs. A total number of 886 sequences were downloaded from NCBI and individually inspected for reasonable sequence length and the presence of the above mentioned conserved motifs. Incomplete sequences or sequences lacking the conserved motifs were not included. The phylogenetic tree (Figure S1) was constructed with the tree building function of Geneious 7.0.6 using the following settings: Alignment type: global alignment with free end gaps, cost matrix: Blosum45, genetic distance model: Jukes-Cantor, tree build method: neighbor joining, outgroop: *Streptomyces exfoliatus* pentalenene synthase (accession number Q55012), gap open penalty: 8, gap extension penalty: 2.

Table S2. Characterised terpene synthases used for HMM analysis.

Substrate	UniProt ID	RefSeq ID	Entry name	Protein name
2-MeGPP	A4FG19	WP_011874125.1	MIBS_SACEN	2-methylisoborneol synthase (EC 4.2.3.118)
2-MeGPP	A3KI17	WP_053126184.1	MIBS_STRA7	2-methylisoborneol synthase (EC 4.2.3.118)
2-MeGPP	Q9F1Y6	WP_011031839.1	MIBS_STRCO	2-methylisoborneol synthase (EC 4.2.3.118)
2-MeGPP	D3KYU2		MIBS_STRLS	2-methylisoborneol synthase (EC 4.2.3.118)
FPP	D9XDR8		AAMS_STRVT	(-)-alpha-amorphene synthase (EC 4.2.3.162)
FPP	Q9UR08		ARIS_ASPTE	aristolochene synthase (EC 4.2.3.9)
FPP	K0K750	WP_015102836.1	BCAS_SACES	(-)-beta-caryophyllene synthase (EC 4.2.3.57)
FPP	Q6WP50		BOT2_BOTFU	presilphiperfolan-8-beta-ol synthase (EC 4.2.3.74)
FPP	E4N7E5		CEABS_KITSK	(+)-corvol ether synthase (EC 4.2.3.163)
FPP	I1S104	XP_011319367.1	CLM1_GIBZE	longiborneol synthase (EC 4.2.3.-)
FPP	A8NE23	XP_001832925.1	COP3_COPC7	alpha-muurolene synthase (EC 4.2.3.125)
FPP	A8NU13	XP_001836356.1	COP4_COPC7	(+)-sativene synthase (EC 4.2.3.129)
FPP	Q9K499	WP_011030119.1	CYC1_STRCO	epi-isozizaene synthase (EC 4.2.3.37)
FPP	Q9X839	WP_011030632.1	CYC2_STRCO	geosmin synthase (EC 4.1.99.16)
FPP	B5GS26	WP_003954606.1	DCADS_STRC2	(-)-delta-cadinene synthase (EC 4.2.3.97)
FPP	D9XD61	WP_003994861.1	EAES_STRVT	7-epi-alpha-eudesmol synthase (EC 4.2.3.169)
FPP	D2B747		ECUB_STRRD	4-epi-cubebol synthase (EC 4.2.3.170)
FPP	S0DX56		EREMS_GIBF5	(+)-eremophilene synthase (EC 4.2.3.164)
FPP	A9FZ87	WP_012241161.1	EREMS_SORC5	(+)-eremophilene synthase (EC 4.2.3.164)
FPP	M4VQY9	XP_747153.1	FMAA_ASPFU	beta-trans-bergamotene synthase (EC 4.2.3.-)
FPP	C7PLV2		GCAS_CHIPD	(-)-gamma-cadinene synthase (EC 4.2.3.62)
FPP	B1W019		GCOA_STRGG	(+)-caryolan-1-ol synthase (EC 4.2.1.138)
FPP	B2J4A4	WP_012410187.1	GERAS_NOSP7	germacrene A synthase (EC 4.2.3.90)
FPP	E8W6C7	WP_014153723.1	GERS_STRFA	(+)-(1(10)E,4E,6S,7R)-germacradien-6-ol synthase
FPP	A0A1L7U8F2		GUDIS_FUSMA	(1R,4R,5S)-(-)-guaia-6,10(14)-diene synthase (EC 4.2.3.165)
FPP	A0A1L7VZE7		GUDIS_FUSPR	(1R,4R,5S)-(-)-guaia-6,10(14)-diene synthase (EC 4.2.3.165)
FPP	E4MYY0	WP_014133196.1	HCS_KITSK	(2Z,6E)-hedycaryol synthase (EC 4.2.3.187)
FPP	Q55012		PENA_STREX	pentalenene synthase (EC 4.2.3.7)
FPP	E3VWJ0		PNTA_STRAE	pentalenene synthase (EC 4.2.3.7)
FPP	B5H7H3	WP_005320742.1	PRISS_STRE2	pristinol synthase (EC 4.2.3.182)
FPP	P0DL13		PRO1_ARMGA	protoillud-6-ene synthase (EC 4.2.3.135)
FPP	Q03471		PRX2_PENRO	aristolochene synthase (EC 4.2.3.9)
FPP	B6H063	XP_002557473.1	PRX2_PENRW	aristolochene synthase (EC 4.2.3.9)
FPP	Q82IY4	WP_010984429.1	PTLA_STRAW	pentalenene synthase (EC 4.2.3.7)
FPP	B5HDJ6	WP_005317515.1	SEDS_STRE2	selina-4(15),7(11)-diene synthase (EC 4.2.3.181)
FPP	A7NH01	WP_012119179.1	TMUUS_ROSCS	(+)-T-muurolol synthase (EC 4.2.3.98)
FPP	B5GW45	WP_003956090.1	TMUUS_STRC2	(+)-T-muurolol synthase (EC 4.2.3.98)
FPP	Q82RR7	WP_010981512.1	TPC1_STRAW	avermitilol synthase (EC 4.2.3.96)
FPP	P13513		TRI5_FUSSP	trichodiene synthase (EC 4.2.3.6)
GGPP	Q96WT2		ACS_PLEBE	aphidicolan-16-beta-ol synthase (EC 4.2.3.42)
GGPP	C9K2Q3		BSC8_ALTBR	fusicocca-2,10(14)-diene synthase (EC 4.2.3.43)
GGPP	A0A0H5BN57		CLDD_STRCP	(12E)-labda-8(17),12,14-triene synthase (EC 4.2.3.193)
GGPP	C9K1X5		COTB2_STRMJ	cyclooctat-9-en-7-ol synthase (EC 4.2.3.146)
GGPP	Q9UVY5		CPSKS_GIBFU	ent-kaur-16-ene synthase (EC 4.2.3.19)
GGPP	O13284		CPSKS_PHASA	ent-kaur-16-ene synthase (EC 4.2.3.19)
GGPP	Q9AJE3	WP_043911627.1	CYC2_KITGR	terpentetriene synthase (EC 4.2.3.36)
GGPP	M1V9Q0		DTCYA_STRSQ	nephthenol synthase (EC 4.2.3.149)
GGPP	M1VDX3		DTCYB_STRSQ	nephthenol synthase (EC 4.2.3.149)
GGPP	A2PZA5		FC1_PHOAM	fusicocca-2,10(14)-diene synthase (EC 4.2.3.43)
GGPP	S0EA85		GA6_GIBF5	ent-kaurene synthase (EC 4.2.3.19)
GGPP	B5GRC8	WP_003954347.1	LATES_STRC2	labda-7,13(16),14-triene synthase (EC 4.2.3.192)
GGPP	A1C8C3	XP_001276070.1	OPHFS_ASPCL	ophiobolin F synthase (EC 4.2.3.145)
GGPP	B2DBF0		PADC1_PHOAM	phyllocladan-16-alpha-ol synthase (EC 4.2.3.45)
GGPP	Q5KSN4		PMDS_STREO	ent-pimara-9(11),15-diene synthase (EC 4.2.3.31)
GGPP	A0A2L0VXR0		PS_CLIPA	premutilin synthase (EC 4.2.3.-)
GGPP	A0A0H5BN61		RMND_STRAQ	(12E)-labda-8(17),12,14-triene synthase (EC 4.2.3.193)
GGPP	A0A1B4XBG5		SDNA_SORAA	cycloaraneosene synthase (EC 4.2.3.-)
GGPP	P0DPK6	WP_030261827.1	SPVIS_STRVB	spiroviolene synthase (EC 4.2.3.158)
GGPP	I2N045	WP_006348711.1	TSUKU_STRT9	tsukubadiene synthase (EC 4.2.3.159)
GPP	B5GMG2	WP_003952918.1	CNSA_STRC2	1,8-cineole synthase (EC 4.2.3.108)
GPP/FPP	D5SL78	WP_003963519.1	LNSA_STRC2	(R)-linalool synthase (EC 4.2.3.26)
HexPP	O34707	WP_004399076.1	YTPB_BACSU	tetraprenyl-beta-curcumene synthase (EC 4.2.3.130)

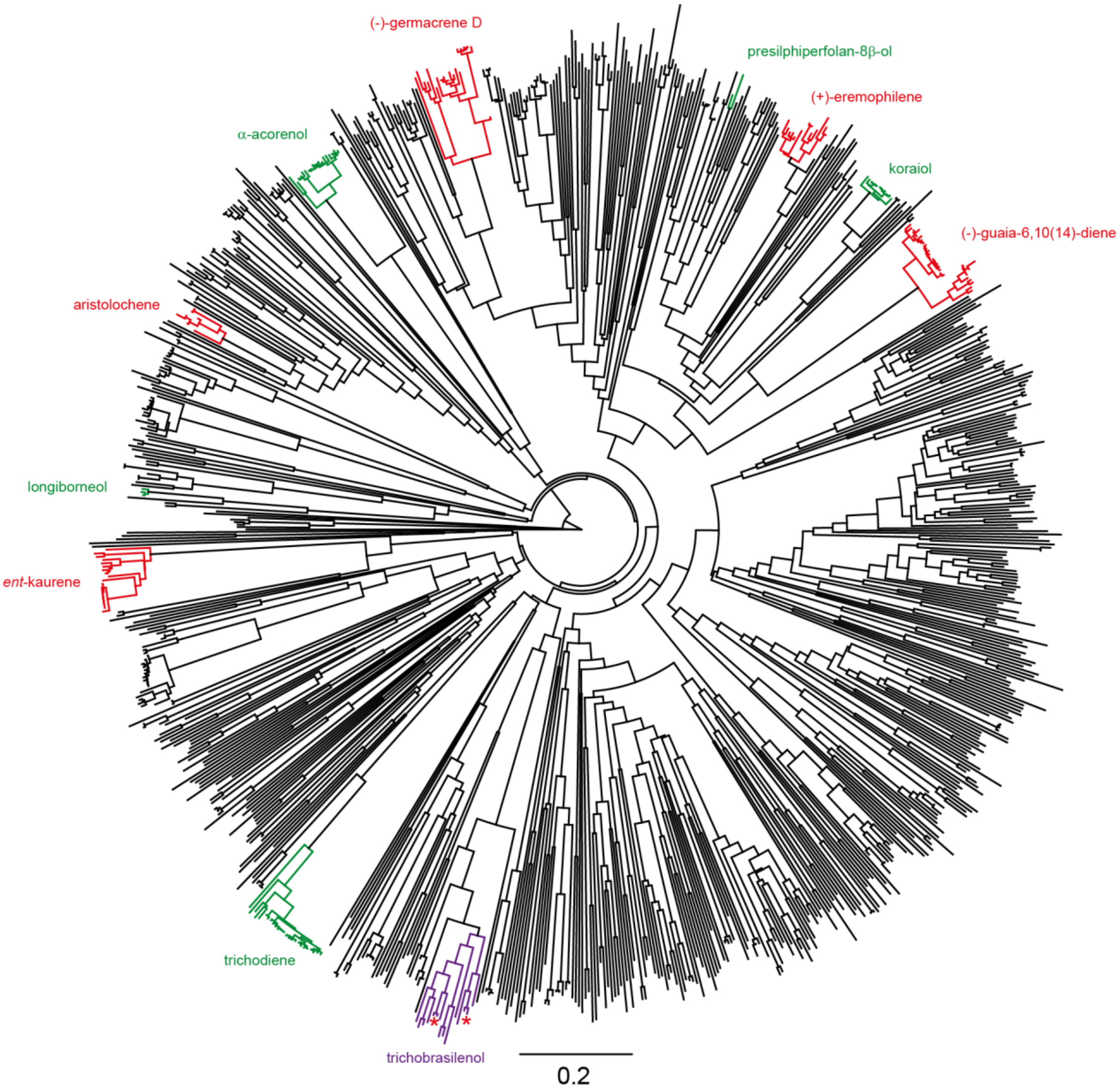

Figure S1. Phylogenetic tree of TaTC6 and 886 putative fungal terpene synthases. The TaTC6 sequence is shown in Figure S4 and is published under accession number LC484924. Characterised enzymes[1-8] and their closest homologues with putatively the same function are shown in green and red. The branch of trichobrasilenol synthases is shown in purple. The enzymes characterised in this study are indicated by red asterisks. The scale bar displays substitutions per site.

In vivo functional analysis of TaTC6

To perform in vivo functional analysis of TaTC6, we used a heterologous expression system with an *A. oryzae* M-2-3 host[9] and a pUARA2 vector.[10] Primers *taTC6*_KpnI_Fw and *taTC6*_KpnI_Rv (Table S1) were used to amplify the region from the predicted start codon of the *taTC6* gene to about 300 bp downstream of the predicted stop codon. The amplified DNA fragment was cloned into a pT7Blue T-vector (Takara Bio Inc., Tokyo, Japan) yielding plasmid pT7Blue_taTC6 and the sequence of the cloned gene was confirmed by sequencing. Plasmid pT7Blue_taTC6 was digested with KpnI and the target DNA fragment containing the TaTC6 sequence was ligated with KpnI-digested pUARA2. The resulting plasmid pUARA2_taTC6 was introduced into the *A. oryzae* M-2-3 host using the protoplast-polyethylene glycol method.[11]

The transformant harboring pUARA2_taTC6 (*A. oryzae* M-2-3/pUARA2_taTC6) was pre-cultured in PDB medium (2.4% Potato Dextrose Broth (Difco)) at 30°C for 3 days before growing in a 500 mL Erlenmeyer flask containing 100 mL of CD + starch medium (0.3 % $NaNO_3$, 0.2 % KCl, 0.1 % KH_2PO_4, 0.05 % $MgSO_4{\cdot}7H_2O$, 0.002 % $FeSO_4{\cdot}7H_2O$, 2.0 % glucose, 2.0 % soluble starch, 1.0 % polypeptone) and incubated on a rotary shaker (160 rpm) at 30 °C. After 3 days of cultivation, acetone was added to the culture broth (2:1, v/v), followed by filtration and concentration under reduced pressure until most of the acetone was removed. The resulting aqueous phase was extracted with ethyl acetate and the organic layer was evaporated to dryness. GC-MS analysis of the crude extract revealed that the transformant *A. oryzae* M-2-3/pUARA2_taTC6 produces a specific metabolite, which is not detectable in the broth of *A. oryzae* M-2-3/pUARA2 (Figure S2).

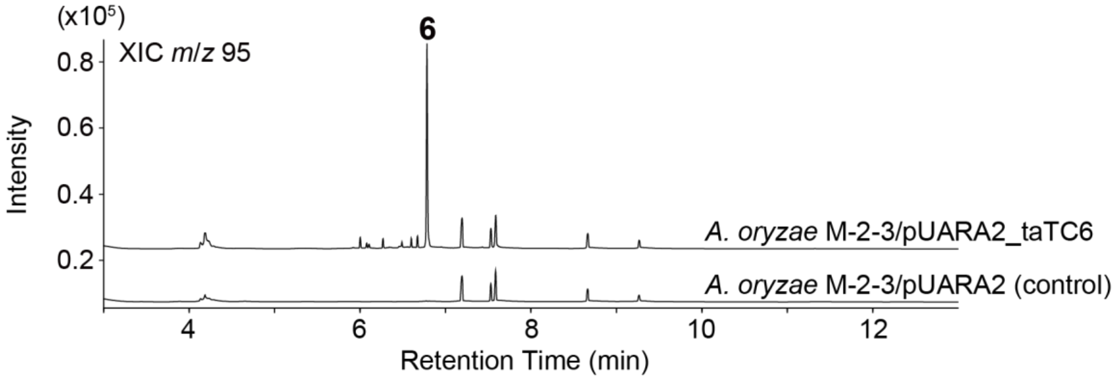

Figure S2. Total ion chromatograms of culture extracts from A) *A. oryzae* M-2-3/pUARA2_taTC6 showing production of **6**, and B) *A. oryzae* M-2-3/pUARA2 (negative control). For the EI mass spectrum of **6** cf. Figure S6.

GC/MS

GC/MS analyses (Bonn) were performed using a system of a 7890B GC fitted with a HP5-MS fused silica capillary column (30 m, 0.25 mm i. d., 0.50 μm film) coupled with a 5977A mass detector (Agilent, Santa Clara, CA, USA). GC parameters were 1) inlet pressure: 77.1 kPa, He at 23.3 mL min^{-1}, 2) injector temperature: 250°C, 3) injection volume: 2 μL, 4) temperature program: 5 min at 50 °C, then increasing at 5 °C min^{-1} to 320 °C, 5) 60 s valve time, and 6) carrier gas: He at 1.2 mL min^{-1}. MS parameters were 1) source: 230 °C, 2) transfer line: 250 °C, 3) quadrupole: 150 °C and 4) electron energy: 70 eV. Retention indices (*I*) were determined in comparison to a homologous series of *n*-alkanes (C_7-C_{40}).

GC/MS analyses (Tokyo) were performed using a GCMS-QP2020 system equipped with a SH-Rxi-5MS column (30 m, 0.25 mm i. d., 0.25 μm film) (Shimadzu, Kyoto, Japan). Injector temperature: 230°C, temperature program: 3 min at 50 °C, then increasing at 10 °C min^{-1} to 130 °C, then increasing at 20 °C min^{-1} to 280 °C.

Cloning of the *T. atroviride* TaTC6 cDNA from *A. oryzae* M-2-3/pUARA2_taTC6

The freeze-dried mycelium (50 mg) of *A. oryzae* M-2-3/pUARA2_taTC6 was ground with a mortar and pestle. Sepasol®-RNA I Super G (nacalai tesque, Kyoto, Japan) was added while freezing with liquid nitrogen. After the mycelium was thawed, chloroform was added, mixed and then allowed to stand at room temperature for 2 minutes, followed by centrifugation. (12,000 rpm, 4°C, 10 min). The aqueous layer was purified using Direct-zol™ RNA MiniPrep (ZYMO RESEARCH, Irvine, USA) according to the manufacturer's instructions. The DNase I treatment was performed on-column; 50 μl of RQ1 RNase-free DNase (Promega, Tokyo, Japan) was added followed by incubation for 1 h at 37°C. For the reverse transcription, the Prime Script® RT-PCR Kit (Takara Bio Inc., Tokyo, Japan) and the Oligo dT Primer packed together with the kit were used. To prepare a total RNA reverse transcription product, 1 µg of total RNA was used.

Primers (RT_*taTC6*_Nhis_Fw and RT_*taTC6*_Nhis_Rv) were used to amplify the region from the predicted start codon to the predicted stop codon of the *taTC6* gene from the total RNA reverse transcription product of the *A. oryzae* M-2-3/pUARA2_taTC6 transformant by PCR. Homologous recombination between the *taTC6* gene and BamHI–HindIII-digested pHis8[12] by the SLiCE method[13] yielded the plasmid pHIS8_taTC6. The nucleotide sequence of the *taTC6* gene has been deposited in the DDBJ/EMBL/GenBank nucleotide sequence databases with the accession number LC484924 and is shown in Figure S4.

Gene expression and protein purification

For gene expression *E. coli* BL21(DE3) was transformed with plasmid pHIS8_taTC6. A preculture of this transformant was grown in terrific broth (TB, 1.2 % tryptone, 2.4 % yeast extract, 0.5 % glycerol, fill up to 900 mL with H_2O, autoclave at 121 °C for 20 min, add 100 mL TB salt solution (0.17 M KH_2PO_4, 0.72 M K_2HPO_4)) containing kanamycin (50 µg mL^{-1}) overnight at 37 °C with shaking at 160 rpm. The preculture was used to inoculate larger culture volumes (1 mL L^{-1} culture) in TB containing kanamycin which were then grown for ~ 4 h until OD_{600} of 0.4 – 0.6 was reached. The cultures were chilled on ice and IPTG solution (400 mM, 1mL L^{-1}) was added to induce protein expression. Expression was carried out at 18 °C with shaking at 160 rpm overnight. The cultures were centrifuged (3.600 x g, 36 min) to separate the cells from the medium. The supernatant was discarded and the cells were resuspended in binding buffer (10 mL L^{-1} culture, 20 mM Na_2HPO_4, 500 mM NaCl, 20 mM imidazole, 1 mM $MgCl_2$, pH = 7.4, 4 °C). Cell lysis was performed using ultra sonication (6 x 1 min) and the resulting suspension was spun down (14.610 x g, 2 x 7 min) to remove the cell debris. The supernatant was filtrated and transferred to a Ni^{2+}-NTA affinity column (Super Ni-NTA, Generon, Slough, UK). Undesired proteins were washed off the column with binding buffer (2 x 10 mL L^{-1} culture), the desired, His-tagged protein was eluted with elution buffer (10 mL L^{-1} culture, 20 mM Na_2HPO_4, 500 mM NaCl, 500 mM imidazole, 1 mM $MgCl_2$, pH = 7.4, 4 °C).

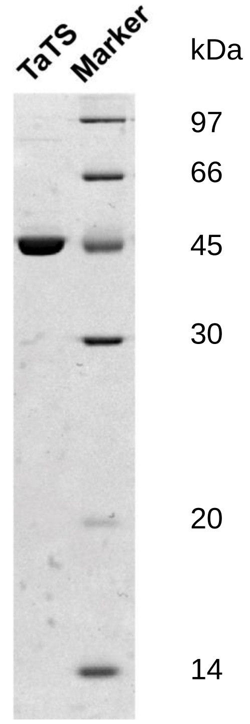

Figure S3. SDS-PAGE of purified recombinant TaTC6.

A)

ATGGGTCAACCCACGACAACTAGCCTTTTTATGAGAGATGTTATGTTTCATCGTATGACTGGCACTAG
TCAGGCCGTGAATGATGTCGCCACGCTATCTGGGGAGCGTAGAGAGATTATCAGAAGAGCTCTCAACA
AGAAAATTCTTGTGCCAAATATTTTGGAACTGATGCCTGCATGGCCTAGTGAATTCCAGCCAAATATC
GATGAGGTCAATGTCGAAATTGATGAATGGCTAAAAACTGTAAATGTTGCAAAAGAGAAAAAACTAAA
ACACAGAGCTCGGGGAAATTATACCTTATTAGCAGGCATTTACTATCCACACTGCAGAAAAGAAAAGA
TGTTGGCTCTCTCTCAGTTTCTGTACTGGATATTCTTCTGGGACGACGAAATTGATACCGGTGGAGAG
CTCACTGAAGATAGAGAAGGCACGATATTATGTTGTGCTGAAACTAATAAGTGCATCAACGATTGTCT
CGGCCCCGAACCCAACTATACACCTCCTCCGGGCTCCCGAGGCACCGTGGAGATGCTCTATCCCATTC
TGAGAGACCTCCGGGCTGGTCTTAGTCCTGTTTCCACTATGCGGTTGAAACAAGAGCTCCACGACTAC
GTCAATGGGGTAAAAAACCAGCAAAAGGTTCGCCAAGAGGATCATCTTCCAAATCCTTGGGACCATTT
CCAAATGCGTGTCGATGATGTTGGAGTCATCCCAAGTATCACTCAGAATGAATATGCCATGGACTTTA
CGCTTCCAGATTGGATTCGCAGGCATGAAGCTATGGAAGAGATTGTCCTGCAATGCACTAAGCTGACA
ATTCTATTGAATGAGATCTTGTCTCTGCAAAAAGAATTCCGAGTTTCTCAACTAGAGAATCTTTGCCT
GCTGTTCATGAATACTTATGATATGTCTATTGAACAATCCATTCACAAAGTCCTTGGTCTTCTGAAAG
ATCACTACAAGATCTGTATTGAGGCTGAAGCCAGATTGCCTTGGAGCACCACTGATGAGAAGCTTAAC
AATAATATTCGTGAGTATATCCGTGGCTGCCAAAGACTGGCAACGGGTACTGCTTGCTGGAGCTACAA
CTGCGAAAGATATTTCAAATTGAGCCAGCTGAATGATCAACAAGAGCTACTGTTGGATTTATCGCGTA
CATGA

B)

MGQPTTTSLFMRDVMFHRMTGTSQAVNDVATLSGERREIIRRALNKKILVPNILELMPAWPSEFQPNI
DEVNVEIDEWLKTVNVAKEKKLKHRARGNYTLLAGIYYPHCRKEKMLALSQFLYWIFFWDDEIDTGGE
LTEDREGTILCCAETNKCINDCLGPEPNYTPPPGSRGTVEMLYPILRDLRAGLSPVSTMRLKQELHDY
VNGVKNQQKVRQEDHLPNPWDHFQMRVDDVGVIPSITQNEYAMDFTLPDWIRRHEAMEEIVLQCTKLT
ILLNEILSLQKEFRVSQLENLCLLFMNTYDMSIEQSIHKVLGLLKDHYKICIEAEARLPWSTTDEKLN
NNIREYIRGCQRLATGTACWSYNCERYFKLSQLNDQQELLLDLSRT

Figure S4. A) Nucleotide sequence and B) amino acid sequence of TaTC6. Conserved motifs were identified by alignment with classical fungal terpene synthases (Figure S5) and are highlighted in yellow.

```
             1        10        20        30        40        50        60
             |        |         |         |         |         |         |
  TaTC6     ------------------MTGTSQTVDDVATLSGERREVIRRALNKKILVPYILELMPAW
 Q9UR08     ---------------------------------------MKKPNGTNGASSSLEPPPSTF
 Q6WP50     MAIPALEPQLHDADTSSNNMSSNSTDSGYDTNSTTPLEKSEKPNTQELKQQQLDPKRPPF

  TaTC6     -------------PSEFQPNIDEINVKIDEW-LKTVNVSKEKKIKHRAQGNYTLLAGIYY
 Q9UR08     -------------QPLCHPLVEEVSKEVDGYFLQHWNFPNEKARKKFVAAGFSRVTCLYF
 Q6WP50     VRVPDLFGSIMSTKPVVNPNYFAAKARGDRWIARVMNFNKAVAARN-SKVDLCFLASMWA

  TaTC6     PHCRKEKMLALSQFLYWIFFWDDEIDTGGELTEDREGTILCCAETNKCIDDCLGPEPNYT
 Q9UR08     PKALDDRIHFACRLLTVLFLIDDLLEYMSF----EEGSAYNEKLIPISRGDVLPDR----
 Q6WP50     PDAPEDRLVMMLDWNHWVFLFDDQFDEGHL----KEDPAAAAEEVKQTIAIMGGNAPRYT

  TaTC6     PPPGSRGTVEMLYPILRDLRAGLSPVSTMRLKQELHDYVNGVRNQQKVRQEDHLPN---P
 Q9UR08     -------SIPVEY-IIYDLWESMRA-HDREMADEILE---PVFLFMRAQTDRTRAR---P
 Q6WP50     AE-----SNPIRY-VFQQCWDRLKAVSSQEMQQRWIDQHKRYFDQLLVQVDQQVGGENFT

  TaTC6     WD---HFQMRVDDVGVIPSITQNEYAMEFTL-PDWIRRHEAMEEIVLQCTKLTILLNEIL
 Q9UR08     MGLGGYLEYRERDVGKELLAALMRFSMGLKLSPSELQR---VREIDANCSKHLSVVNDIY
 Q6WP50     RDVEAYMDLRRGTIGVYPAISLSEYGAGVNV-PQHVYDHPSLQECMKVSADLVTLVNDVL

  TaTC6     SLQKEF--------RVSQLENLCLLFMNTYDMSIEQSIHKVLGLLKDHYKICIEAEARLP
 Q9UR08     SYEKELYTSKTAHSEGGILCTSVQILAQEADVTAEAAKRVLFVMCREWELRHQLLVARL-
 Q6WP50     SYRKDL--------ELGVDHNLMSLLMQRDNLSAQQAVDVIGDMVNECYRRWYLALAELP

  TaTC6     WSTTDEKLNNNIREYIRGCQRLATGTACWSYNCERYFKLSQLNDQQELLLDLSRT-----
 Q9UR08     --SAEGLETPGLAAYVEGLEYQMSGNELWSQTTLRY----------SVVVD---------
 Q6WP50     --SYGEKIDYNVMKFVEICRAVAQGNLYWSFQTGRYLGPEGHEVHETGIMYLPPAANLVV

  TaTC6     -
 Q9UR08     -
 Q6WP50     A
```

Figure S5. Alignment of TaTC6 with classical fungal terpene synthases (Q9UR08: *Aspergillus terreus* NIH2624, aristolochene synthase; Q6WP50: *Botrytis cinerea*, presilphiperfolan-8-beta-ol synthase).[4,7]

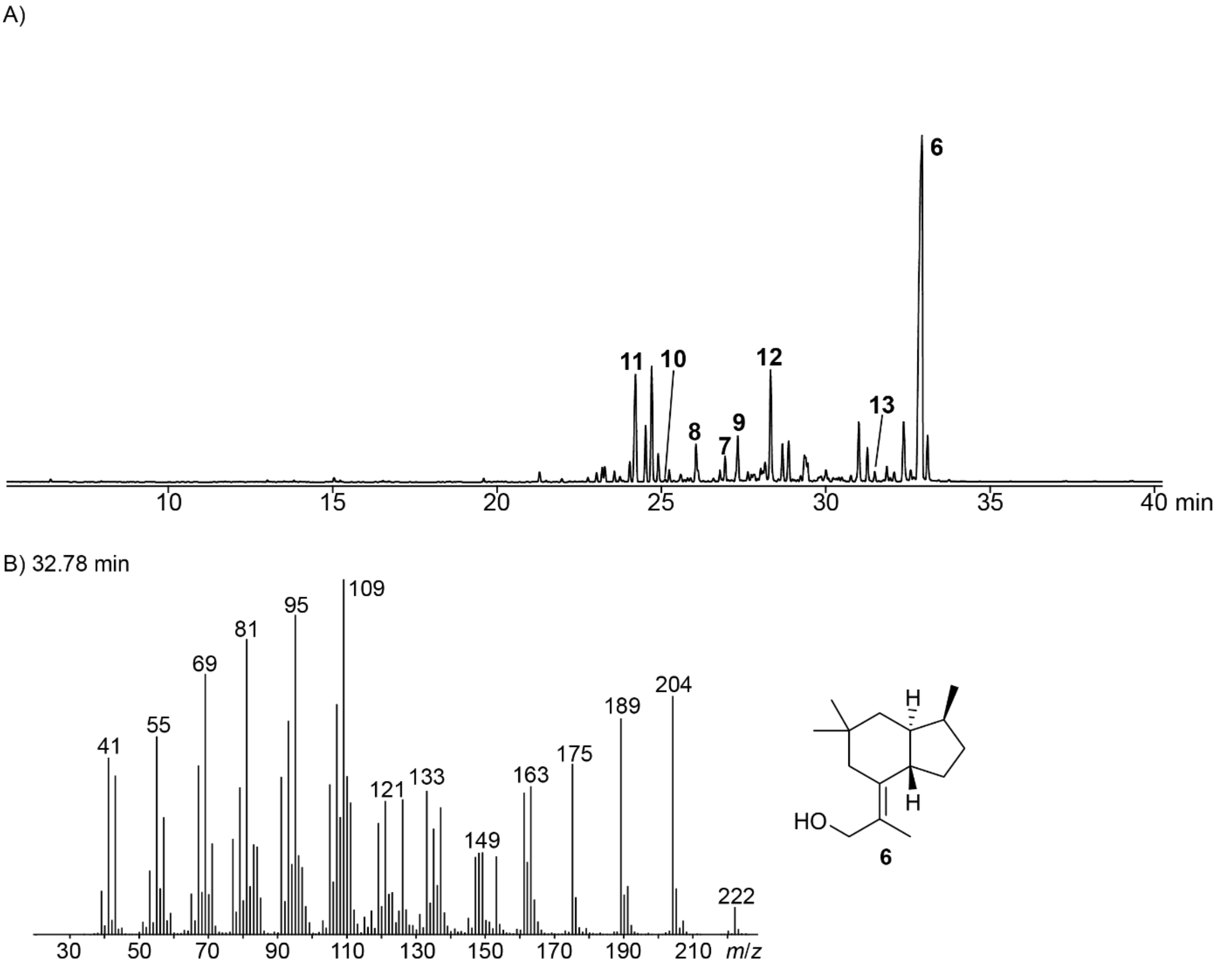

Figure S6. A) Total ion chromatogram of a crude extract from an incubation of FPP with TaTC6 (see Table S5), B) EI mass spectrum of trichobrasilenol (**6**).

Incubation experiments with recombinant TaTC6

Incubation experiments were performed using freshly prepared enzyme solutions and FPP (final concentration 0.5mg mL^{-1}) dissolved in substrate buffer (25 mm NH_4HCO_3). The enzyme solution was diluted with an equal volume of incubation buffer (50 mM TRIS, 10 mM $MgCl_2$, 20 % glycerol, pH 8.2). The mixtures were shaken at 28 °C for 3 h. Small scale incubations using 1 mg substrate were extracted with 200 µL hexane, the organic phase was directly subjected to GC-MS. Incubation experiments for the preparative production of bungoene using 60 mg FPP were extracted with hexane (3 x 150 mL), the combined organic layers were dried with $MgSO_4$, concentrated under reduced pressure and subjected to column chromatography on SiO_2 (pentane:Et_2O, 2:1). The desired fractions were combined and evaporated to give trichobrasilenol as colourless oil.

NMR spectroscopy

NMR spectra were recorded on a Bruker (Billerica, MA, USA) Avance III HD Prodigy (500 MHz) or an Avance III HD Cryo (700 MHz) NMR spectrometer. Spectra were referenced against solvent signals (^{1}H-NMR, residual proton signals: C_6D_6 δ = 7.16, ^{13}C-NMR: C_6D_6 δ = 128.06).[14]

Trichobrasilenol, 2-((1*S*,3a*S*,7a*S*,*E*)-1,6,6-trimethyloctahydro-4*H*-inden-4-ylidene)propan-1-ol (6). Colourless oil. Yield: 10.5 mg (0.05 mmol, 45 %). R_f (pentane:Et_2O, 5:1) = 0.3. $[\alpha]_D^{20}$ = +6.5° (*c* 1.05, C_6D_6). HRMS (APCI): *m*/*z* = 223.2055 (calc. for $[C_{15}H_{27}O]^+$ 223.2056). GC (HP5-MS): *I* = 1710. MS (EI, 70 eV): *m*/*z* (%) = 41 (50), 43 (45), 53 (18), 55 (56), 57 (33), 67 (48), 69 (73), 71 (25), 77 (27), 79 (41), 81 (83), 83 (25), 91 (44), 93 (60), 95 (90), 105 (42), 107 (65), 109 (100), 119 (31), 121 (37), 126 (38), 133 (40), 135 (30), 137 (36), 147 (22), 148 (22), 149 (22), 153 (22), 161 (40), 163 (42), 175 (48), 189 (61), 204 (67), 222 (8), see Figure S6. NMR spectral data are given in Table S3 and Figures S7–S13.

Table S3. NMR spectral data of trichobrasilenol (**6**) in C_6D_6 recorded at 298 K.

C[a]		^{1}H[b]	^{13}C[b]
1	CH_2	1.30 (ddd, $^2J_{H,H}$ = 12.7, $^3J_{H,H}$ = 3.9, $^4J_{H,H}$ = 1.8, 1 H, H_α)	41.2
		1.10 (t, $^2J_{H,H}$ = 12.8, 12.8, 1 H, H_β)	
2	CH	1.57 (dddd, $^3J_{H,H}$ = 12.7, 12.6, 8.5, 4.0, 1 H)	45.6
3	CH	1.88 (m, 1 H)	32.1
4	CH_2	1.97 (m, 1 H, H_α)	33.8
		1.01 (m, 1 H, H_β)	
5	CH_2	2.00 (m, 1 H, H_β)	31.6
		1.52 (m, 1 H, H_α)	
6	CH	1.83 (ddd, $^3J_{H,H}$ = 12.3, 12.3, 5.3, 1 H)	48.1
7	C_q	–	127.9
8	CH_2	4.21 (d, $^2J_{H,H}$ = 11.2, 1 H, H_α)	65.2
		3.89 (d, $^2J_{H,H}$ = 11.2, 1 H, H_β)	
9	C_q	–	136.4
10	CH_2	2.34 (dd, $^2J_{H,H}$ = 13.7, $^4J_{H,H}$ = 1.5 , 1 H, H_α)	44.2
		1.52 (m, 1 H, H_β)	
11	C_q	–	33.2
12	CH_3	0.82 (s, 3 H)	26.5
13	CH_3	0.93 (s, 3 H)	32.4
14	CH_3	1.86 (s, 3 H)	17.9
15	CH_3	0.78 (d, $^3J_{H,H}$ = 7.2, 3 H)	18.2

[a] Carbon numbering as shown in Scheme 2 of main text. [b] Chemical shifts δ in ppm, multiplicity: s = singlet, d = doublet, t = triplet, m = multiplet, coupling constants *J* are given in Hertz.

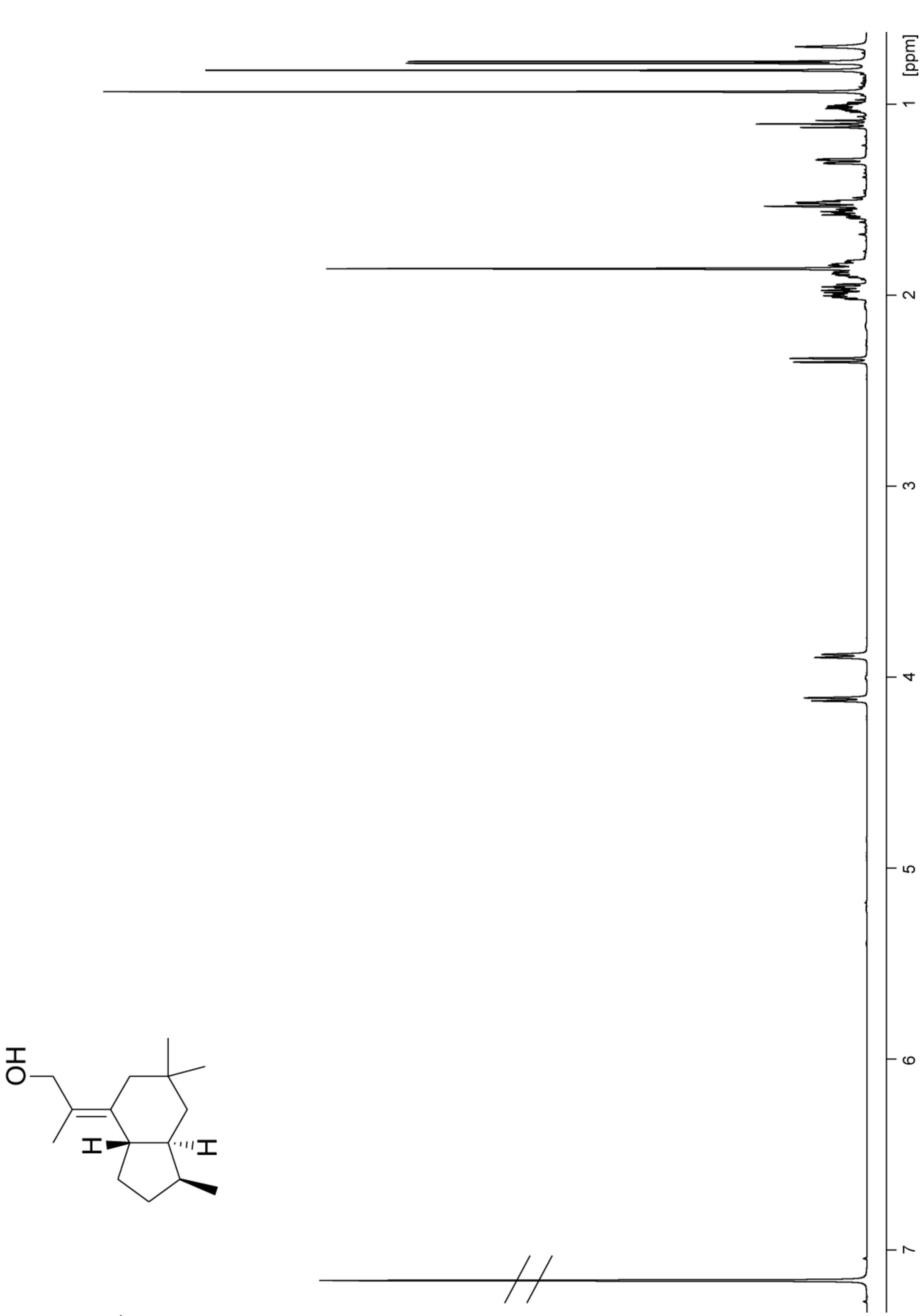

Figure S7. ^{1}H-NMR spectrum of **6** (700 MHz, C_6D_6).

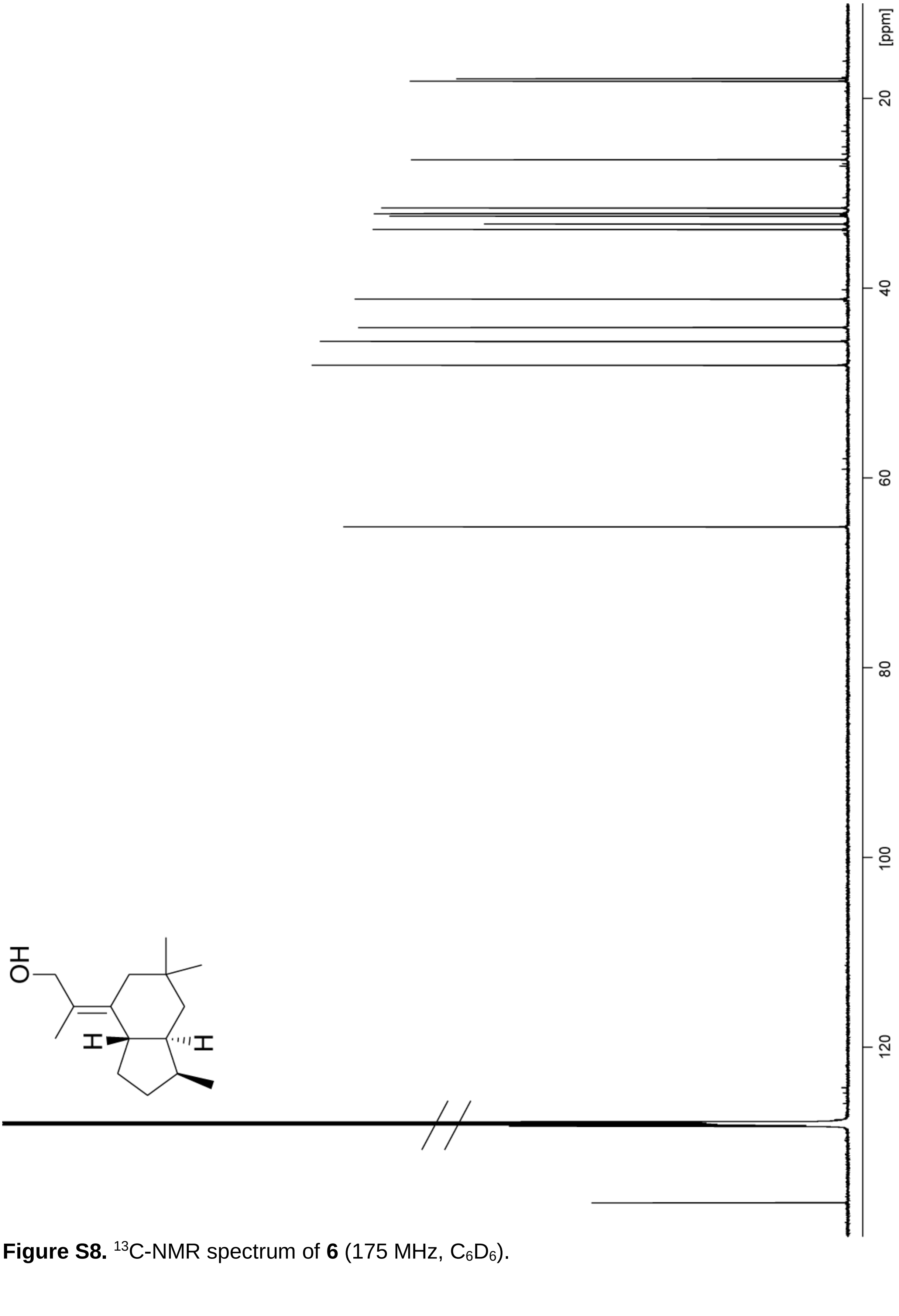

Figure S8. ^{13}C-NMR spectrum of **6** (175 MHz, C_6D_6).

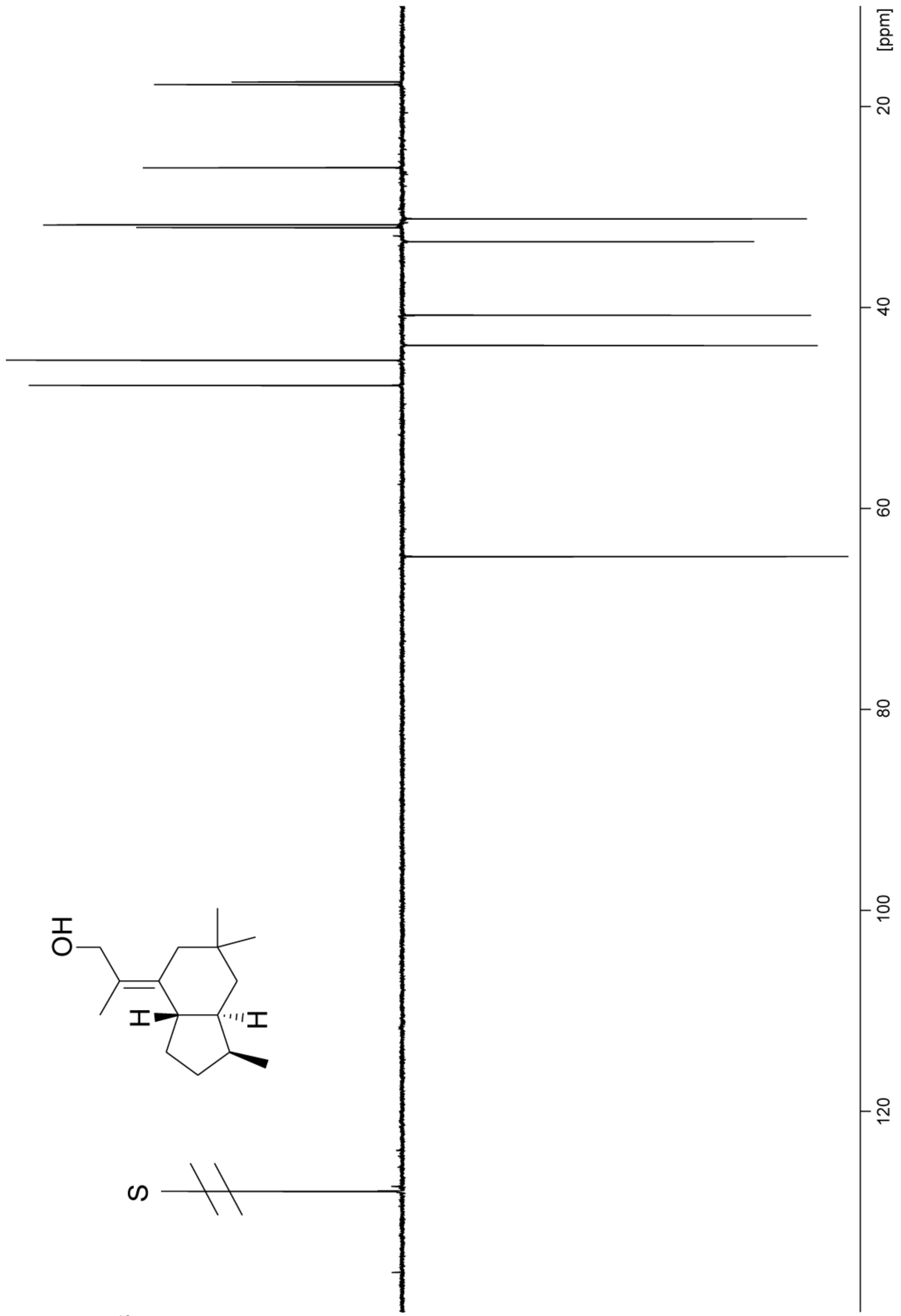

Figure S9. ^{13}C-DEPT 135 spectrum of **6** (175 MHz, C_6D_6), S = residual solvent signal.

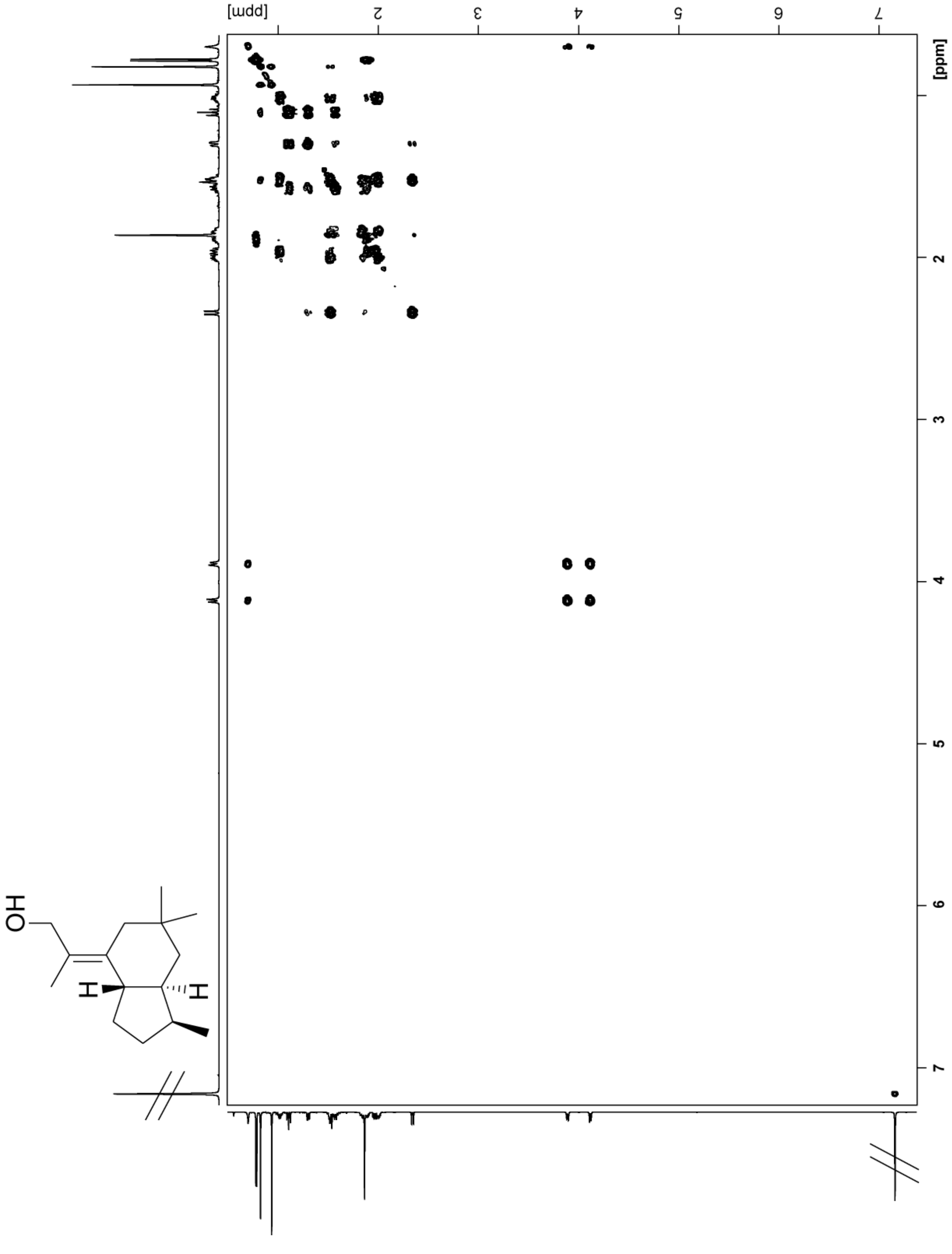

Figure S10. ${}^{1}H,{}^{1}H$-COSY spectrum of **6** (C_6D_6).

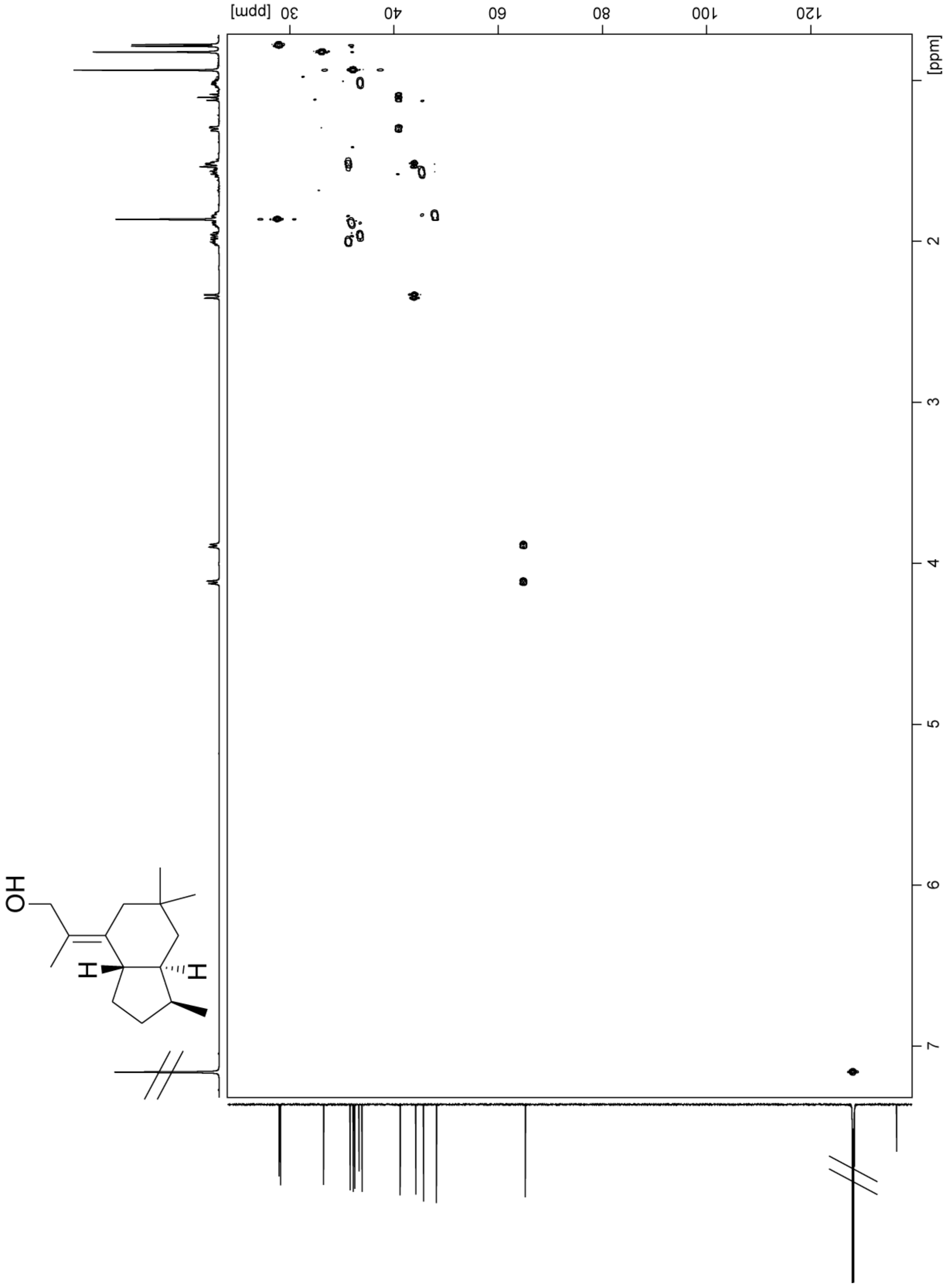

Figure S11. HSQC spectrum of **6** (C_6D_6).

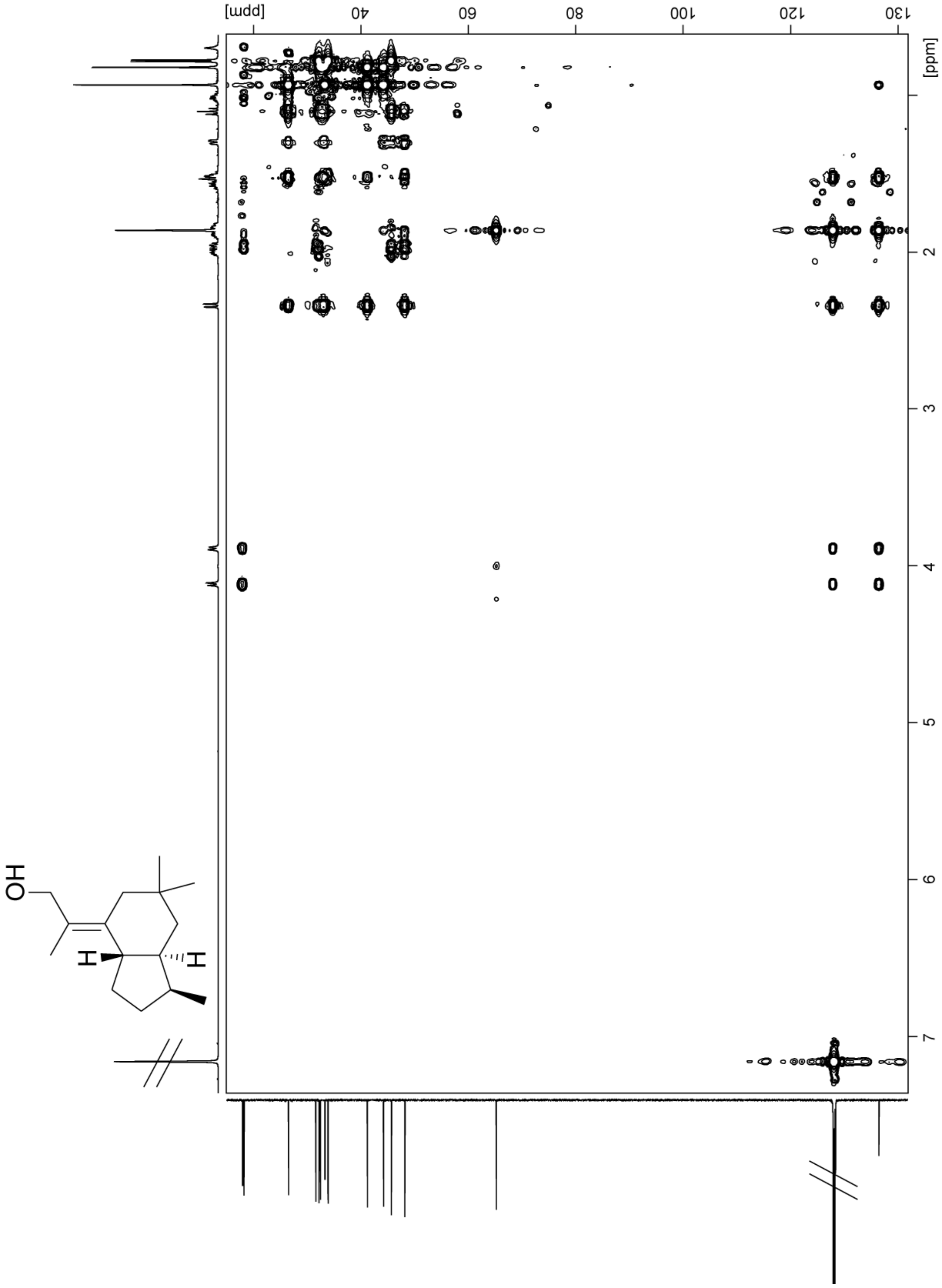

Figure S12. HMBC spectrum of **6** (C_6D_6).

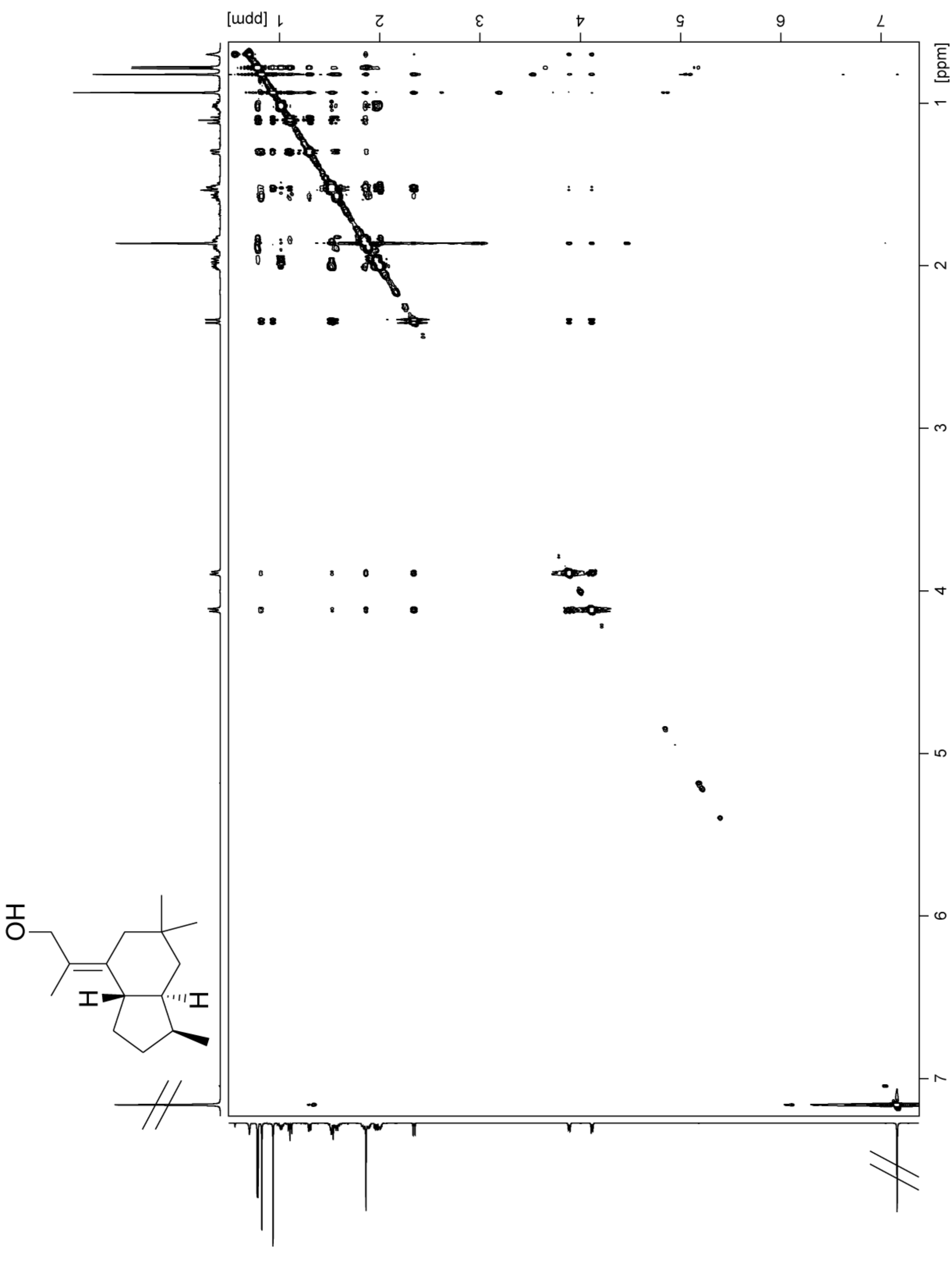

Figure S13. NOESY spectrum of **6** (C_6D_6).

```
                                                  1        10        20        30        40        50        60
                                                  |        |         |         |         |         |         |
          LC484924 (Trichoderma atroviride FKI-3849)   MGQPTTTSLFMRDVMFHRMTGTSQAVNDVATLSGERREIIRRALNKKILVPNILELMPAW
              XP_006969402 (Trichoderma reesei QM6a)   MDSLWSIVVTIRDIALKRTVGTDHLHKAAAEVLDERKDVIRRALNKKVLVPDILALMPEW
    XP_024759911 (Trichoderma asperellum CBS 433.97)   ------------------MPGVIQITDNAAKPFDEREEVIRRALNKKVLVPNILELMPAW
    XP_013949081 (Trichoderma atroviride IMI 206040)   ------------------MTGTSQTVDDVATLSGERREVIRRALNKKILVPYILELMPAW
XP_024748823 (Trichoderma citrinoviride TUCIM 6016)   MDSLWSVIDFIRDIAVKHTVGSSHIHKAAAEAFNERKDVIRRALNKKVLIPDILAFMPAW
     XP_024770660 (Trichoderma harzianum CBS 226.95)   MDQLRSLVFFLRDVALKHTLGTSHTKNDTTEIFDERKEVIKRVLNKKVLVPDILALMPAW
        OTA00344 (Trichoderma parareesei CBS 125925)   MDSLWSIVVFIRDIALKRTVGTDHLHKAAAEVLDERKDVIRRALNKKVLVPDILALMPDW
           XP_013961713 (Trichoderma virens Gv29-8)   MDQLRSLVFFIRDVAFKHTLGNNHPKKDAPKIFDERKEVIRRALNKKVLVPDILSLMPAW

          LC484924 (Trichoderma atroviride FKI-3849)   PSEFQPNIDEVNVEIDEWLKTVNVAKEKKLKHRARGNYTLLAGIYYPHCRKEKMLALSQF
              XP_006969402 (Trichoderma reesei QM6a)   PSEFQPDIDEVNLEIDEWLKTVKVAEEKKAKHRARGNYTLLAGIYYPHCKKDKLLVLSQF
    XP_024759911 (Trichoderma asperellum CBS 433.97)   PSEFQPDIDEINVKVDEWLKTVNVAKEKKLKHRTRGNYTLLAGIYYPHCKKEKMLALSQF
    XP_013949081 (Trichoderma atroviride IMI 206040)   PSEFQPNIDEINVKIDEWLKTVNVSKEKKIKHRAQGNYTLLAGIYYPHCRKEKMLALSQF
XP_024748823 (Trichoderma citrinoviride TUCIM 6016)   PSEFQPDVDEINLELDEWLQSVNVAEEKKAKHRARGNYTLLAGIYYPHCKKDKLLVLSQF
     XP_024770660 (Trichoderma harzianum CBS 226.95)   ISEFQPDIDEINLEIDEWLKTVNVAEEKKAKHRARGNYTLLTGIYYPHCKKDKMLVLSQF
        OTA00344 (Trichoderma parareesei CBS 125925)   PSEFQPDIDEVNLEIDEWLKTVKVAEEKKAKHRARGNYTLLAGIYYPHCKKDKLLVLSQF
           XP_013961713 (Trichoderma virens Gv29-8)   PSEFQPDIDEINLEIDEWLKTVNVAEEKKAKHRARGNYTLLTGVYYPHCKKDKMLVLSQF

          LC484924 (Trichoderma atroviride FKI-3849)   LYWIFFWDDEIDTGGELTEDREGTILCCAETNKCINDCLGPEPNYTPPPGSRGTVEMLYP
              XP_006969402 (Trichoderma reesei QM6a)   LYWIFFWDDEIDTGGELTHDRQGTLQCCAETNKCIDDCLGPNPNYSPPPGSRGTVEMFYP
    XP_024759911 (Trichoderma asperellum CBS 433.97)   LYWIFFWDDEIDTGGDLTEDREGTILCCAETNKCIDDCLGPSPNYTPPSGSRGTVEILYP
    XP_013949081 (Trichoderma atroviride IMI 206040)   LYWIFFWDDEIDTGGELTEDREGTILCCAETNKCIDDCLGPEPNYTPPPGSRGTVEMLYP
XP_024748823 (Trichoderma citrinoviride TUCIM 6016)   LYWIFFWDDEIDTGGELTHDRQGTLQCCAETNKCIDDCLGPNPNYTPPPGARGTVEMLYP
     XP_024770660 (Trichoderma harzianum CBS 226.95)   LYWIFFWDDEIDTGGELTHDREGTLQCCAETNKCVDDCLGPNPNYTPPPGSRGTVEMFYP
        OTA00344 (Trichoderma parareesei CBS 125925)   LYWIFFWDDEIDTGGELTHDRQGTLQCCAETNKCIDDCLGPNPNYSPPPGSRGTVEMFYP
           XP_013961713 (Trichoderma virens Gv29-8)   LYWIFFWDDEIDTGGELTYDREGTLQCCAETNKCIDDCLGPNPCYTPPPGSRGTVEMFYP

          LC484924 (Trichoderma atroviride FKI-3849)   ILRDLRAGLSPVSTMRLKQELHDYVNGVKNQQKVRQEDHLPNPWDHFQMRVDDVGVIPSI
              XP_006969402 (Trichoderma reesei QM6a)   ILRDLRSGLGPVSTERLRQELHDYVNGAANQQGVREEDHLPDPWVHFQMRADDVGVIPSI
    XP_024759911 (Trichoderma asperellum CBS 433.97)   ILRDLRAGLGPVSTMRLKKELHDYVNGVSNQQKVRQEDHLPNPWDHFQMRVDDVGVIPSI
    XP_013949081 (Trichoderma atroviride IMI 206040)   ILRDLRAGLSPVSTMRLKQELHDYVNGVRNQQKVRQEDHLPNPWDHFQMRVDDVGVIPSI
XP_024748823 (Trichoderma citrinoviride TUCIM 6016)   ILRDLRSGLGPVSTERLRKELHDYVNGAANQQKVREEDHLPDPWDHFKMRSDDVGVIPSI
     XP_024770660 (Trichoderma harzianum CBS 226.95)   ILRDLRKGLGPSSTERLRKELHDYINGASTQQHVREADHLPDPWDHFNNRANDVGAIPSI
        OTA00344 (Trichoderma parareesei CBS 125925)   ILRDLRSGLGPVSTERLRQELHDYVNGAANQQGVREEDHLPDPWVHFQMRADDVGVIPSI
           XP_013961713 (Trichoderma virens Gv29-8)   ILRDLRKGLGPVSTERLRKELHDYVNGASNQQRVREEDRLPDPWDHFQMRADDVGVIPSI
```

Figure S14. Alignment of TaTC6 with closely related enzymes from other *Trichoderma* strains.

```
         LC484924 (Trichoderma atroviride FKI-3849)   TQNEYAMDFTLPDWIRRHEAMEEIVLQCTKLTILLNEILSLQKEFRVSQLENLCLLFMNT
             XP_006969402 (Trichoderma reesei QM6a)   TQNEYAMEFELPEWIRRHEAMEEIVLECTKLTILLNEVLSLQKEFRVSQLENLCLLFMNT
   XP_024759911 (Trichoderma asperellum CBS 433.97)   TQNEYAMEFELPDWIRKHEAMEEIVLQCTKLTILLNEILSLQKEFRVSQLENLCLLFMNT
   XP_013949081 (Trichoderma atroviride IMI 206040)   TQNEYAMEFTLPDWIRRHEAMEEIVLQCTKLTILLNEILSLQKEFRVSQLENLCLLFMNT
XP_024748823 (Trichoderma citrinoviride TUCIM 6016)   TQNEYAMEFELPEWIRRHEAMEEIVLECTKLTILLNEVLSLQKEFRVSQLENLCLLFMNT
    XP_024770660 (Trichoderma harzianum CBS 226.95)   TQNEFAMEFELPEWVRRHEAMEEIVLECTKLTILVNEVQSSQKEFRVSQLENLCFLFMNA
       OTA00344 (Trichoderma parareesei CBS 125925)   TQNEYAMEFELPERIRRHEAMEEIVLECTKLTILLNEVLSLQKEFRVSQLENLCLLFMNT
           XP_013961713 (Trichoderma virens Gv29-8)   TQNEYAMEFELPEWIRRHEAMEVIVLECTKLTILLNEVLSLQKEFRDNQLENLCFLFMNT

         LC484924 (Trichoderma atroviride FKI-3849)   YDMSIEQSIHKVLGLLKDHYKICIEAEARLPWSTTDEKLNNNIREYIRGCQRLATGTACW
             XP_006969402 (Trichoderma reesei QM6a)   DNVSIEEAIDKILGLLQEHYEICVAAEARLPWSETDEKLNEDIREYVRGANRLATGTACW
   XP_024759911 (Trichoderma asperellum CBS 433.97)   YNISIEQSINKILGLLKDHYTICTEAEARIPWSTTDEKLNSDIREYIRGCQRLATGTACW
   XP_013949081 (Trichoderma atroviride IMI 206040)   YDMSIEQSIHKVLGLLKDHYKICIEAEARLPWSTTDEKLNNNIREYIRGCQRLATGTACW
XP_024748823 (Trichoderma citrinoviride TUCIM 6016)   DNLSIEEAIGKILDLLQEHYEICVAAEARLPWSETDEKLNEDIREYVRGANRLATGTACW
    XP_024770660 (Trichoderma harzianum CBS 226.95)   NNLSIEEAIDKVLGILKEHYEICVAAEARLPWSKTDEKFNEDLREYVRGCQRLATGTACW
       OTA00344 (Trichoderma parareesei CBS 125925)   DNVSIEEAIDKILGLLQEHYEICVAAEARLPWSETDEKLNEDIREYVRGANRLATGTACW
           XP_013961713 (Trichoderma virens Gv29-8)   YNLSIEDSIDKVLGLLKEHYDICVAAEARLPWSKTDEKLNEDIREYVRGCNRLATGTACW

         LC484924 (Trichoderma atroviride FKI-3849)   SYNCERYFKLSQLNDQQELLLDLSRT-
             XP_006969402 (Trichoderma reesei QM6a)   SYNCERYFKLSQVNEKRELLLDLSYR-
   XP_024759911 (Trichoderma asperellum CBS 433.97)   SYNCERYFKLSQLNNKQELLLDLSRT-
   XP_013949081 (Trichoderma atroviride IMI 206040)   SYNCERYFKLSQLNDQQELLLDLSRT-
XP_024748823 (Trichoderma citrinoviride TUCIM 6016)   SYNCERYFKLSQVNEKRELLLDLSYQ-
    XP_024770660 (Trichoderma harzianum CBS 226.95)   SYNCERYFKLSQLNDKRELLLDYSYQK
       OTA00344 (Trichoderma parareesei CBS 125925)   SYNCERYFKLSQVNEKRELLLDLSYR-
           XP_013961713 (Trichoderma virens Gv29-8)   SYNCERYFKLSQLNDKRELLLDLSYQK
```

Figure S14. Alignment of TaTC6 with closely related enzymes from other *Trichoderma* strains.

Plasmid construction for expression in *Aspergillus oryzae*

Fungal expression plasmid pTAex3 and *Aspergillus oryzae* NSAR1 were kindly provided by Prof. K. Gomi (Graduate School of Agricultural Sciences, Tohoku University) and Prof. K. Kitamoto (Graduate School of Agricultural Sciences, The University of Tokyo). Primers used for plasmid construction are listed in Table S1. PCRs were performed with Q5 polymerase (New England Biolabs, Ipswich, MA, USA) and ligations were performed with In-Fusion® HD cloning kit (Clontech, Saint-Germain-en-Laye, France) according to the manuals provided by the manufacturers. All plasmids were constructed in *Escherichia coli* Stellar (Clontech, Saint-Germain-en-Laye, France) that was grown on LB medium with ampicillin. Transformations of *A. oryzae* were performed with the protoplast–polyethylene glycol method, as reported previously.[15] Candidate transformants were stabilized in proper selective agar medium (M agar with arginine and methionine for *A. oryzae* NSAR1-FPPS-OE: 0.2 % NH_4Cl, 0.1 % $(NH_4)_2SO_4$, 0.05 % KCl, 0.05 % NaCl, 0.1 % KH_2PO_4, 0.05 % $MgSO_4$, 0.002% $FeSO_4/7H_2O$, 2 % glucose, 0.1 % arginine, 0.15 % methionine, 1.5 % agar, pH=5.5; and M agar with methionine for *A. oryzae* NSAR1-FPPS-OE-NC and *A. oryzae* NSAR1-FPPS-OE-TrTS: 0.2 % NH_4Cl, 0.1 % $(NH_4)_2SO_4$, 0.05 % KCl, 0.05 % NaCl, 0.1 % KH_2PO_4, 0.05 % $MgSO_4$, 0.002% $FeSO_4/7H_2O$, 2 % glucose, 0.15 % methionine, 1.5 % agar, pH=5.5) at 30 °C for 3 days, then inoculated in DPY medium (2 % dextrin, 1 % polypeptone, 0.5 % yeast extract, 0.05 % $MgSO_4$, 0.5 % KH_2PO_4) for further analysis.

To enhance the production of sesquiterpenes, the FPP synthase (FPPS) from *Aspergillus nidulans* FGSC A4 was overexpressed in *Aspergillus oryzae* NSAR1. For this purpose, plasmid pAdeA-FPPS, derived from pUC19, was constructed. First, the terminator (700 bp) and promoter regions (1100 bp) from the TEF gene (accession number BAA76296) were cloned into pUC19. The TEF terminator sequence was amplified from gDNA of *A. oryzae* RIB40 using primers TEF_Tmn-Fw and TEF_Tmn-Rv and cloned into pUC19 digested with BamHI and HindIII to yield pUC19-TEF_Tmn. Then the TEF promoter sequence was amplified from gDNA of *A. oryzae* RIB40 using primers TEF_Prm-Fw and TEF_Prm-Rv and cloned into pUC19-TEF_Tmn digested with SacI to yield plasmid pTEF_cst. In the next step, the FPPS gene from *Aspergillus nidulans* FGSC A4 (accession number XP_681281) was amplified from genomic DNA using primers FPPS_Fw and FPPS_Rv and cloned into pTEF_cst digested with SmaI to yield plasmid pTEF-FPPS. In the final step the selection marker AdeA from *A. oryzae* RIB40 (accession number BAC98505) was amplified with primers AdeA_Fw and AdeA_Rv and cloned into pUC19 linearized by PCR using primers pUC19_Fw and pUC19_Rv to yield pUC19-AdeA. The TEF promoter – FPPS – TEF terminator cassette was then amplified from plasmid pTEF-FPPS using primers TEF_2_AdeA-Fw and TEF_2_AdeA-Rv and cloned into pUC19-AdeA digested with SacI to yield the final plasmid pAdeA-FPPS. This plasmid was transformed into *A. oryzae* NSAR1 to give the strain *A. oryzae* NSAR1-FPPS-OE. Plasmid pTAex3-TrTS was constructed for the heterologous expression of the TrTS gene from *Trichoderma reesei* QM6a. For this purpose, the gene (accession number XP_006969402) was amplified from genomic DNA using primers TrTS_Fw and TrTS_Rv and the amplificate was cloned into pTAex3 digested with EcoRI to yield pTAex3-TrTS. This plasmid was transformed into *A. oryzae* NSAR1-FPPS-OE to give *A. oryzae* NSAR1-FPPS-OE-TrTS. For a negative control experiment, the empty vector pTAex3 was also transformed into *A. oryzae* NSAR1-FPPS-OE to generate *A. oryzae* NSAR1-FPPS-OE-NC.

Detection of trichobrasilenol (6) production

A. oryzae NSAR1-FPPS-OE-TrTS was cultivated in DPY medium and incubated for 3 days at 30 °C. The mycelia were separated and dried and extracted with acetone under sonication. The extracts were subjected to GC/MS analysis (Figure S15).

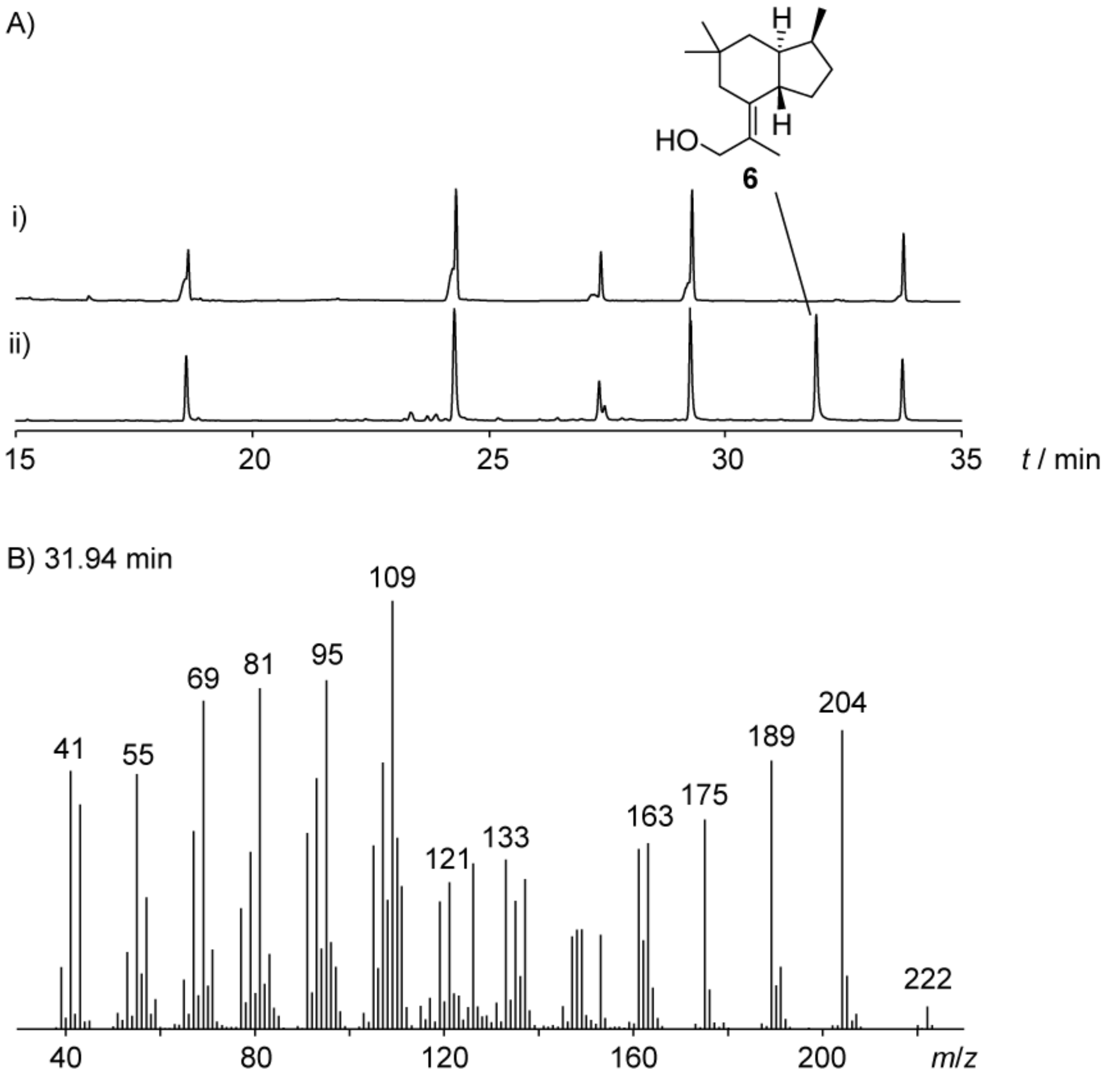

Figure S15. A) GC/MS chromatogram of crude extracts from i) *A. oryzae* NSAR1-FPPS-OE-NC, and ii) *A. oryzae* NSAR1-FPPS-OE-TrTS. B) EI mass spectrum of **6**.

Incubation experiments with isotopically labelled substrates

Isotopic labelling experiments were performed with amounts of ca. 1 mg labelled FPP (or its precursors) in substrate buffer (1 mL), incubation buffer (4 mL), enzyme elution fractions (to 8 mL total volume) with the substrates and enzyme preparations as listed in Table S3. After incubation with shaking at 28 °C for 3 h the products were extracted with C_6D_6 (650 µL) for NMR and GC/MS experiments. The extracts were dried with $MgSO_4$ and analysed by NMR and GC/MS.

Table S4. Isotopic labelling experiments with TaTC6.

entry	substrate(s)	enzyme(s)	results shown in
1	DMAPP + (*E*)-(4-^{13}C,4-^{2}H)IPP[16]	TaTC6 + FPPS[17]	Figure 2
2	DMAPP + (*Z*)-(4-^{13}C,4-^{2}H)IPP[16]	TaTC6 + FPPS[17]	Figure 2
3	(3-^{13}C,2-^{2}H)FPP[18]	TaTC6	Figure 3
4	(2-^{2}H)GPP[19] + (2-^{13}C)IPP[20]	TaTC6 + FPPS[17]	Figure 3
5	(2-^{13}C,1,1-^{2}H^{2})DMAPP[17] + (2-^{13}C)IPP[20]	TaTC6 + FPPS[17]	Figure 3
6	(*R*)-(1-^{13}C,1-^{2}H)IPP[21] + DMAPP	TaTC6 + FPPS[17]	Figure S16
7	(*S*)-(1-^{13}C,1-^{2}H)IPP[21] + DMAPP	TaTC6 + FPPS[17]	Figure S16
8	(1-^{13}C)FPP[22]	TaTC6	Figure S17
9	(2-^{13}C)FPP[22]	TaTC6	Figure S17
10	(3-^{13}C)FPP[22]	TaTC6	Figure S17
11	(4-^{13}C)FPP[22]	TaTC6	Figure S17
12	(5-^{13}C)FPP[22]	TaTC6	Figure S17
13	(6-^{13}C)FPP[22]	TaTC6	Figure S17
14	(7-^{13}C)FPP[22]	TaTC6	Figure S17
15	(8-^{13}C)FPP[22]	TaTC6	Figure S17
16	(9-^{13}C)FPP[22]	TaTC6	Figure S17
17	(10-^{13}C)FPP[22]	TaTC6	Figure S17
18	(11-^{13}C)FPP[22]	TaTC6	Figure S17
19	(12-^{13}C)FPP[22]	TaTC6	Figure S17
20	(9-^{13}C)GPP[19] + IPP	TaTC6 + FPPS[17]	Figure S17
21	(10-^{13}C)GPP[23] + IPP	TaTC6 + FPPS[17]	Figure S17
22	(15-^{13}C)FPP[22]	TaTC6	Figure S17

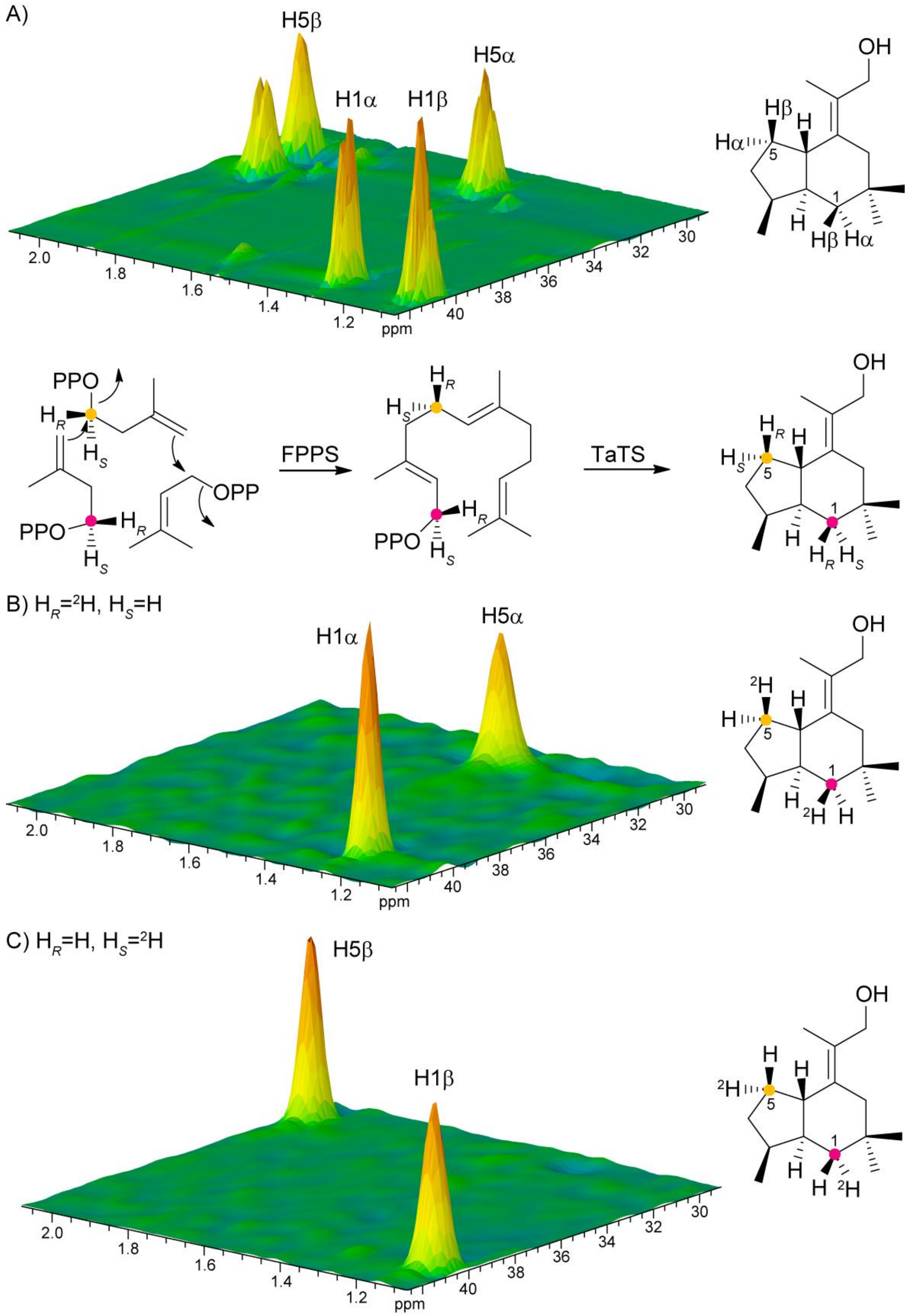

Figure S16. Determination of the absolute configuration of **6** using the FPPS to elongate DMAPP with (*R*)- and (*S*)-(1-^{13}C,1-^{2}H)IPP. Coloured dots indicate ^{13}C-labelling.

Table S5. Side products from conversion of FPP with TaTC6.

compound	*m*/*z*	t_R / min	*I*	*I* (lit)
african-1-ene (**11**)	204	24.25	1362	1356[24]
african-3-ene (**10**)	204	25.11	1393	1391[24]
(*E*)-β-caryophyllene (**8**)	204	26.04	1429	1421[24]
α-humulene (**7**)	204	26.94	1455	1460[24]
2-*epi*-(*E*)-β-caryophyllene (**9**)	204	27.33	1479	1467[24]
isoafricanol (**12**)	222	28.27	1540	1528[25]
pristinol (**13**)	222	31.49	1653	1643[18]
trichobrasilenol (**6**)	222	32.78	1710	–

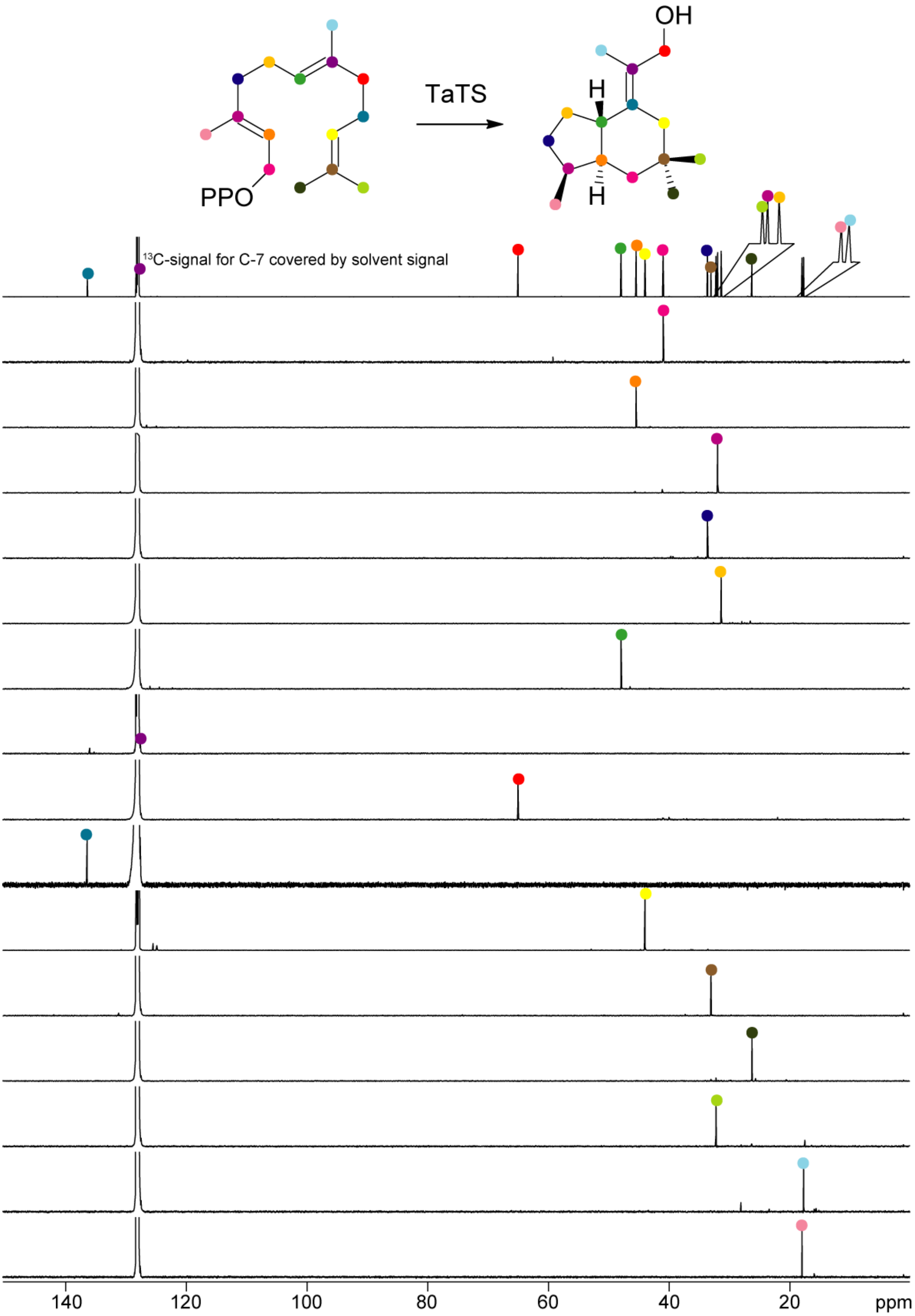

Figure S17. Single ^{13}C-labelling clearly shows the position of each carbon in the sesquiterpene precursor FPP. Coloured dots indicate which signal arises from each carbon, the signal for C-7 (127.9 ppm) is covered by the solvent signal.

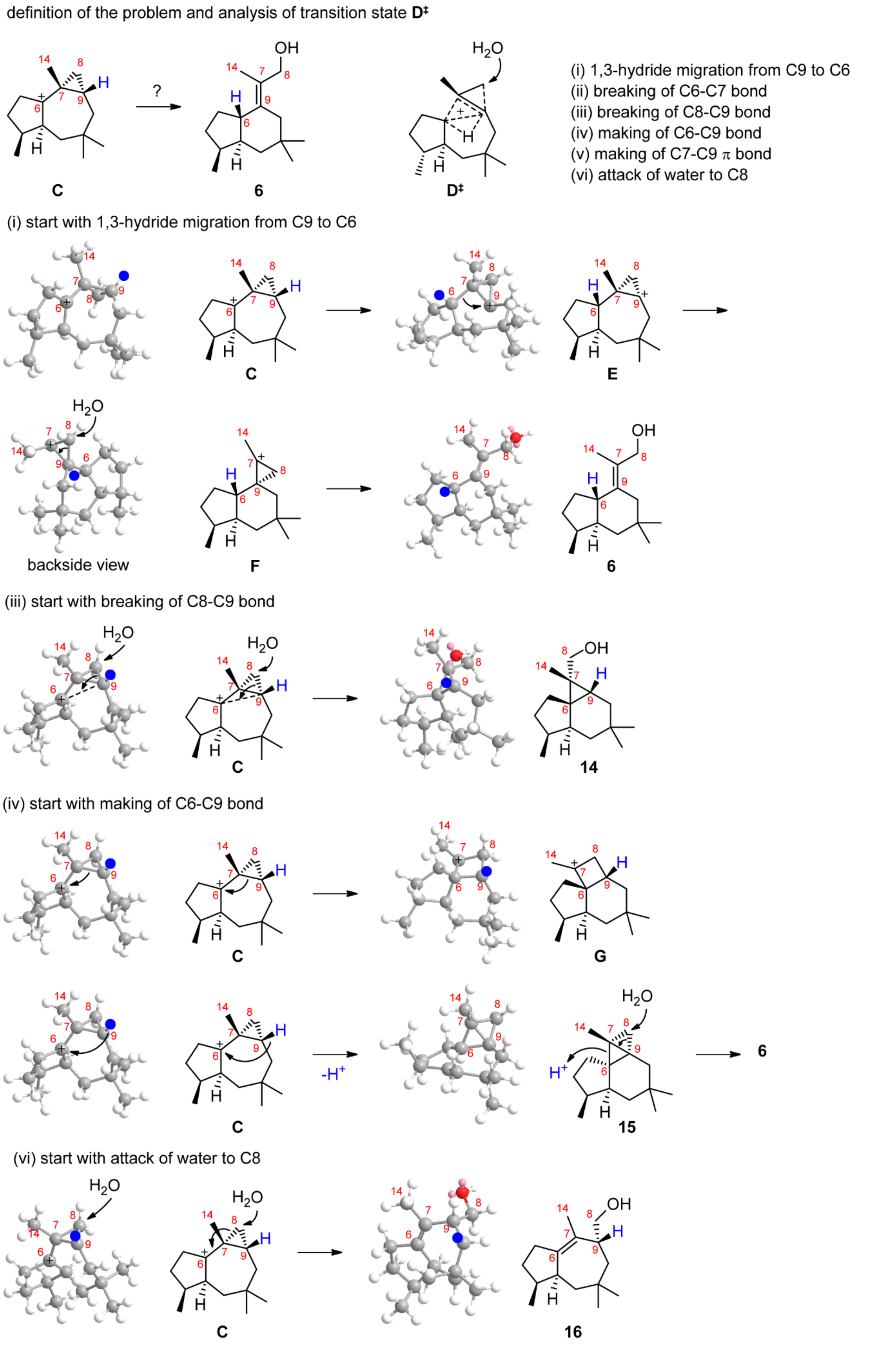

Scheme S2. An attempt to find alternative stepwise processes for the **C**-to-**6** rearrangement.

The formation of **6** requires the rearrangement of **C** taking the results from the labelling experiments into consideration. The relevant carbons C6, C7, C8, C9 and C14 are indicated throughout Scheme S2, and the migrating hydrogen is shown in blue. Transition state **D**‡ gives a mechanistic explanation by a concerted reaction that involves (i) a 1,3-hydride migration from C9 to C6, (ii) breaking of the C6-C7 bond, (iii) breaking of the C8-C9 bond, (iv) making of the C6-C9 bond, (v) making of the C7-C9 π bond, (vi) attack of water to C8. The question addressed in this discussion is: Is there an alternative stepwise process that could maybe better explain the reaction from **C** to **6**? If this is the case, one of the six defined events must be the first elementary step of such a non- or less-concerted mechanism. To find a possible alternative, the six events are systematically tested as first elementary steps. The breaking of the C6-C7 bond (ii) and the making of the C7-C9 π bond (v) are not possible as starting events for a stepwise process and thus do not need to be considered.

(i) Start with the 1,3-hydride migration from C9 to C6. The hypothetical product cation **E** has a cationic centre at the bridgehead carbon C9, making this reaction very unlikely. Its calculated structure (using the MM2 force field function of ChemBio3D Ultra 13.0) demonstrates that all three substituents at the cationic centre C9 point into one hemisphere. Moreover, the calculated structure of E suggests that the hydride migration should undergo with a concerted ring contraction by migration of the C6-C7 to a C6-C9 bond, equal to steps (ii) and (iv). The hypothetical product **F**, a cyclopropyl cation, should undergo immediate ring opening to a more stable allyl cation at C8-C7-C9 that can be captured by water at C8 to form **6**. This analysis of the **C**-to-**6** conversion as a stepwise process involves very unlikely cationic intermediates, but make the overall concerted reaction, theoretically separable into the three discussed elementary steps, very plausible.
(ii) Start with breaking of the C6-C7 bond is not a possible reaction.
(iii) Start with breaking of the C8-C9 bond must proceed with simultaneous making of a C6-C9 bond, as there is no other possibility where the electron density could migrate to. By this process a primary cation at C8 would be formed that could be avoided by simultaneous capture with water, step (vi), to form **14** that is not observed as an enzyme product (but it may be present among the unidentified minor side products). Compound **14** is no longer a reactive intermediate and it is difficult to understand its further conversion into **6**. In conclusion, starting with step (iii) does not result in a good explanation for the formation of **6**.
(iv) Start with making of the C6-C9 bond could proceed like described for (iii). Alternatively, this could be realised by ring expansion of the cyclopropane to **G**, a cation that could potentially be trapped by water to form a sesquiterpene alcohol that was not observed, but may be present among the minor side products of the enzyme. This reaction does not explain the formation of **6** in which a C7=C9 double bond is needed. A third possibility is deprotonation from C9 with formation of a highly strained bicyclobutane moiety in **15**. Its reprotonation at C6 must proceed with reintroduction of the proton abstracted from C9 and would lead to **F** that can rearrange to the allyl cation at C8-C7-C9 discussed in (i), followed by capture with water to **6**.
(v) Start by making of the C7-C9 π bond is not a possible reaction.
(vi) Start with the attack of water to C8 is only possible with simultaneous migration of the electron density in the C7-C8 bond to form a C6-C7 π bond. This reaction can form compound **16** which may be among the minor side products of the enzyme reaction, but it does not explain the formation of **6**.

In conclusion, the concerted reaction from **C** via **D**‡ to **6** gives the best explanation. The theoretical separation into three steps as discussed in (i) makes this reaction more plausible, but the intermediates along this line are instable species that should be avoided as in the concerted mechanism.

References

[1] T. M. Hohn, F. Vanmiddlesworth, *Arch. Biochem. Biophys.* **1986**, *251*, 756.

[2] T. Toyomasu, H. Kawaide, A. Ishizaki, S. Shinoda, M. Otsuka, W. Mitsuhasi, T. Sassa, *Biosci. Biotechnol. Biochem.* **2000**, *64*, 660.

[3] S. P. McCormick, N. J. Alexander, L. J. Harris, *Appl. Environ. Microbiol.* **2010**, *76*, 136.

[4] R. H. Proctor, T. M. Hohn, *J. Biol. Chem.* **1993**, *268*, 4543.

[5] N. L. Brock, K. Huss, B. Tudzynski, J. S. Dickschat, *ChemBioChem* **2013**, *14*, 311.

[6] E.-M. Niehaus, J. Schumacher, I. Burkhardt, P. Rabe, M. Münsterkötter, U. Güldener, J. S. Dickschat, B. Tudzynski, *Front. Microbiol.* **2017**, *8*, 1175.

[7] C. Pinedo, C. M. Wang, J. M. Pradier, B. Dalmais, M. Choquer, P. Le Pecheur, G. Morgant, I. G. Collado, D. E. Cane, M. Viaud, *ACS Chem. Biol.* **2008**, *3*, 791.

[8] I. Burkhardt, T. Siemon, M. Henrot, L. Studt, S. Rösler, B. Tudzynski, M. Christmann, J. S. Dickschat, *Angew. Chem. Int. Ed.* **2016**, *55*, 8748.

[9] T. Fujii, H. Yamaoka, K. Gomi, K. Kitamoto, C. Kumagai, *Biosci. Biotechnol. Biochem*. **1995**, *59*, 1869.

[10] K. Tagami, C. Liu, A. Minami, M. Noike, T. Isaka, S. Fueki, Y. Shichijo, H. Toshima, K. Gomi, T. Dairi, H. Oikawa, *J. Am. Chem. Soc*. **2013**, *135*,1260.

[11] A. Watanabe, Y. Ono, I. Fujii, U. Sankawa, M. E. Mayorga, W. E. Timberlake and Y. Ebizuka, *Tetrahedron Lett.*, **1998**, *39*, 7733.

[12] J. M. Jez, J. L. Ferrer, M. E. Bowman, R. A. Dixon, J. P. Noel, *Biochemistry* **2000**, *39*, 890.

[13] Y. Okegawa, K. Motohashi, *Biochem. Biophys. Reports* **2015**, *4*, 148.

[14] G. R. Fulmer, A. J. M. Miller, N. H. Sherden, H. E. Gottlieb, A. Nudelman, B. M. Stoltz, J. E. Bercaw, K. I. Goldberg, *Organometallics* **2010**, *29*, 2176.

[15] K. Gomi, Y. Iimura, S. Hara, *Agric*. *Biol*. *Chem*. **1987**, *51*, 2549.

[16] L. Lauterbach, J. Rinkel, J. S. Dickschat, *Angew. Chem. Int. Ed.* **2018**, *57*, 8280.

[17] P. Rabe, J. Rinkel, B. Nubbemeyer, T. G. Köllner, F. Chen, J. S. Dickschat, *Angew. Chem. Int. Ed.* **2016**, *55*, 15420.

[18] T. A. Klapschinski, P. Rabe, J. S. Dickschat, *Angew. Chem. Int. Ed.* **2016**, *55*, 10141.

[19] G. Bian, J. Rinkel, Z. Wang, L. Lauterbach, A. Hou, Y. Yuan, Z. Deng, T. Liu, J. S. Dickschat, *Angew. Chem. Int. Ed.* **2018**, *57*, 15885.

[20] J. Rinkel, L. Lauterbach, J. S. Dickschat, *Angew. Chem. Int. Ed.* **2019**, *58*, 452.

[21] J. Rinkel, J. S. Dickschat, *Org. Lett.* **2019**, *21*, 2426-2429.

[22] P. Rabe, L. Barra, J. Rinkel, R. Riclea, C. A. Citron, T. A. Klapschinski, A. Janusko, J. S. Dickschat, *Angew. Chem. Int. Ed.* **2015**, *54*, 13448.

[23] T. Mitsuhashi, J. Rinkel, M. Okada, I. Abe, J. S. Dickschat, *Chem. Eur. J*. **2017**, *23*, 10053.

[24] R. P. Adams, *Identification of Essential Oil Components by Gas Chromatography/Mass Spectrometry*, Allured, Carol Stream, 2009.

[25] P. Rabe, M. Samborskyy, P. F. Leadlay, J. S. Dickschat, *Org. Biomol. Chem.* **2017**, *15*, 2353.
